# Constructing gene regulatory networks using epigenetic data

**DOI:** 10.1038/s41540-021-00208-3

**Published:** 2021-12-09

**Authors:** Abhijeet Rajendra Sonawane, Dawn L. DeMeo, John Quackenbush, Kimberly Glass

**Affiliations:** 1 Channing Division of Network Medicine, Brigham and Women’s Hospital, Boston, MA USA; 2 Harvard Medical School, Boston, MA USA; 3 Center for Interdisciplinary Cardiovascular Sciences, Division of Cardiovascular Medicine, Brigham and Women’s Hospital, Boston, MA USA; 4 Department of Biostatistics, Harvard Chan School of Public Health, Boston, MA USA

**Keywords:** Computational biology and bioinformatics, Regulatory networks, Molecular biology, Reverse engineering

## Abstract

The biological processes that drive cellular function can be represented by a complex network of interactions between regulators (transcription factors) and their targets (genes). A cell’s epigenetic state plays an important role in mediating these interactions, primarily by influencing chromatin accessibility. However, how to effectively use epigenetic data when constructing a gene regulatory network remains an open question. Almost all existing network reconstruction approaches focus on estimating transcription factor to gene connections using transcriptomic data. In contrast, computational approaches for analyzing epigenetic data generally focus on improving transcription factor binding site predictions rather than deducing regulatory network relationships. We bridged this gap by developing SPIDER, a network reconstruction approach that incorporates epigenetic data into a message-passing framework to estimate gene regulatory networks. We validated SPIDER’s predictions using ChIP-seq data from ENCODE and found that SPIDER networks are both highly accurate and include cell-line-specific regulatory interactions. Notably, SPIDER can recover ChIP-seq verified transcription factor binding events in the regulatory regions of genes that do not have a corresponding sequence motif. The networks estimated by SPIDER have the potential to identify novel hypotheses that will allow us to better characterize cell-type and phenotype specific regulatory mechanisms.

## Introduction

Gene regulation is a complex process that includes transcription factors binding and recruiting the transcriptional machinery to the regulatory regions of their target genes^1,2^. The regulatory connections from transcription factors to their target genes can be summarized in a gene regulatory network. Quantitative analysis and comparison of gene regulatory networks supports the characterization of regulatory processes^3,4^, and can provide insights into how cells develop, respond to environmental perturbations^5,6^, and are altered by disease^7,8^.

The construction of gene regulatory networks is a fundamental problem in computational biology^9–11^. Experimental high-throughput DNA binding experiments, such as ChIP-seq^12^, can identify genome-wide, context-specific transcription factor binding sites. Although data from these assays can be used to identify which transcription factors bind within the regulatory regions of genes, they are limited both by their cost and the fact that they are only able to cover a small number of transcription factors due to a lack of good antibodies. This has led to the development of many computational approaches for gene regulatory network reconstruction. Most existing methods focus on estimating relationships between transcription factors and genes exclusively using expression data^13–17^, although some combine expression with other omics data, such as computationally predicted transcription factor binding site locations^18–20^. For example, PANDA (Passing Attributes between Networks for Data Assimilation)^18^ is a multi-omic network reconstruction algorithm that uses message passing to integrate predicted transcription factor binding information with protein–protein interaction and gene co-expression data in order to estimate gene regulatory networks. PANDA has been applied to a wide range of biological problems, including the study of human diseases^7,8,18^, tissues^3,4^, and cell lines^21–23^. Several databases have also begun curating experimental and computational evidence regarding gene regulatory relationships^24–27^, but these sources are not reconstruction algorithms and thus are not designed to reconstruct a network de novo from newly generated, context-specific data.

Gene regulatory networks are challenging to model due to the multi-faceted nature of the regulatory process. For example, transcription factors often work together by forming protein complexes. This leads to instances in which a member of a transcription factor complex regulates a target gene even without a corresponding binding site in the regulatory region of that gene^2^. In addition, the epigenetic state of a cell—i.e., the set of changes to a cell’s genome that do not impact its DNA sequence, including its three-dimensional chromatin structure^28^—influences which regions of the genome are open and accessible, thus impacting transcription factor binding and, consequently, gene regulation. Chromatin accessibility assays, such as DNase-seq^29^ and ATAC-seq^30^, can identify regions of open chromatin that are bound by proteins such as transcription factors, but these assays do not provide the identity of the bound factors. Instead, the potential genomic locations of transcription factors are generally computationally estimated using DNA recognition sequences, called motifs^31–33^.

Transcription factor binding site prediction methods generally work by identifying all the locations in the genome that match a given transcription factor motif, scoring each identified motif location, and benchmarking by assessing how well those scores predict in vivo ChIP-seq transcription factor binding. Motif scores often incorporate information regarding DNA accessibility to improve their ability to predict transcription factor binding sites^34–41^. However, the focus on improving performance using an initial set of motif locations means that these types of methods do not assess instances where a transcription factor binds to the DNA in the absence of a corresponding recognition sequence (motif). Furthermore, these algorithms are exclusively designed and optimized to predict the genomic locations of transcription factor binding rather than deducing gene regulatory network relationships.

On one hand, although there are many algorithms that have been developed for gene regulatory network reconstruction, these approaches are almost exclusively based deducing a network using gene expression data, and do not incorporate epigenetic data. On the other hand, pipelines designed to predict transcription factor binding using epigenetic data are not designed to model network relationships. Here we propose a method that bridges this gap by reconstructing a gene regulatory network based on information exchange between epigenetically accessible motifs. SPIDER (Seeding PANDA Interactions to Derive Epigenetic Regulation) overlaps transcription factor motif locations with epigenetic data (open chromatin locations) and then applies the same message-passing algorithm used by PANDA to construct a gene regulatory network. We applied SPIDER to DNase-seq data for six human cell lines and evaluated the predicted networks using independently derived ChIP-seq data. We find that SPIDER networks are significantly more accurate than those derived from the output of existing epigenetic pipelines. Importantly, we also show that SPIDER’s unique approach of melding epigenetic data with message passing allows for the detection of potential co-regulatory events. An implementation of SPIDER is available at: https://github.com/kimberlyglass/spider.

## Results

### SPIDER: Seeding PANDA Interactions to Derive Epigenetic Regulation

**Fig. 1:**
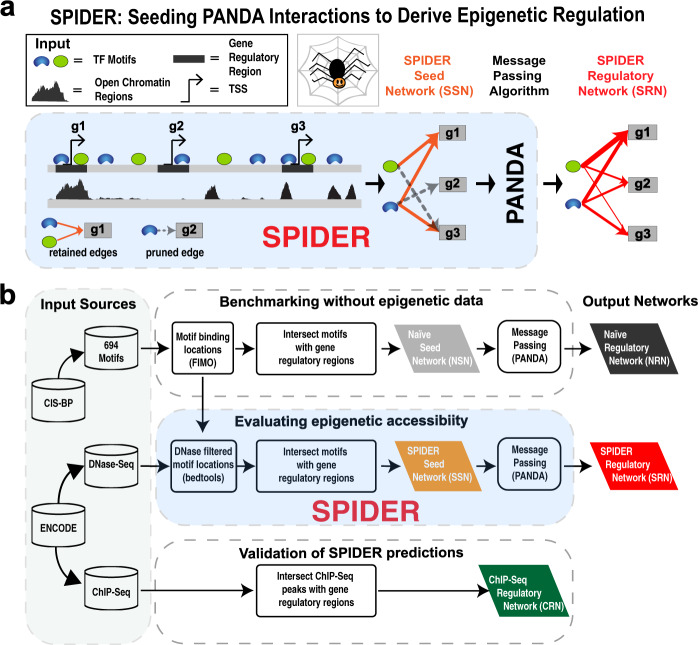
Overview of SPIDER and evaluation pipeline. **a** Schematic of the SPIDER network reconstruction approach. **b** Overview of the pipeline we used to evaluate SPIDER, including input data sources, key algorithmic steps, and output networks assessed. TF transcription factor, TSS transcriptional start site.

To bridge the gap between computational approaches that use epigenetic data to predict transcription factor binding sites and network reconstruction approaches that model the relationships between transcription factors and genes, we developed SPIDER as a method to reconstruct a gene regulatory network using epigenetic data. SPIDER identifies transcription factor motifs found in accessible chromatin regions, uses this information to identify an initial “seed” network, and then applies message passing to harmonize connections across all the transcription factors and genes (Fig. 1a). The input to SPIDER includes the genomic location of (1) transcription factor motifs, defined by position weight matrices mapped onto the DNA^42^, (2) open chromatin regions, based on epigenetic data, and (3) gene regulatory regions, which can be defined based on proximity to transcriptional start sites. SPIDER first constructs a “seed” network between transcription factors and target genes by intersecting transcription factor motif locations with open chromatin and gene regulatory regions. An edge in this seed network represents a transcription factor that has a motif location that overlaps with both an open chromatin region and the target gene’s regulatory region. Next, the weights of edges in this seed network are degree-normalized to emphasize connections to high degree transcription factors and genes (see Methods); by definition, these transcription factors and genes are associated with more open chromatin regions and are therefore more likely to be active players in the regulatory process. The structure of this network is then optimized using the PANDA message-passing algorithm^18^. The output of SPIDER is a bipartite, complete network with weighted edges representing the likelihood of regulatory relationships between all transcription factors and all target genes. For a more detailed description of SPIDER, see Methods.

We tested SPIDER using data for six human cell lines (Table 1). For the input to SPIDER we used (1) transcription factor motif locations derived from mapping transcription factor position weight matrices from Cis-BP^43^ to the hg19 genome using FIMO^42^, (2) open chromatin regions defined in narrow-Peak DNase-seq data files from ENCODE, and (3) regulatory regions, defined as 2 kilobase (kb) windows centered around the transcriptional start sites of genes based on RefSeq annotations^44^ (Fig. 1b). For each cell line we estimated two epigenetically informed networks: an initial “seed” network constructed from intersecting transcription factor binding motif locations with open chromatin and gene regulatory regions (orange parallelogram in Fig. 1b) and a final SPIDER regulatory network estimated by applying message passing to the seed network (red parallelogram). We also estimated two reference networks: an epigenetically “naïve” seed network of transcription factor to gene associations derived from intersecting motif locations with regulatory regions (gray parallelogram), and a final naïve regulatory network derived by applying message passing to the naïve seed network (black parallelogram). All of these networks include regulatory associations between 687 transcription factors and 27,090 target genes. Finally, we created six “gold standard” validation networks, one for each of the six cell lines, by taking the intersection of peaks from cell-line-specific ChIP-seq experiments and gene regulatory regions (green parallelogram). It should be noted that the dimensions of these ChIP-seq networks vary based on the transcription factors assayed in each cell line (Table 1); however, the number of target genes in the networks (27,090) is always the same. In total we have fourteen reconstructed networks—two (SPIDER seed network, SPIDER regulatory network) for each of the six cell lines as well as the “naïve” seed and regulatory networks—and six “gold standard” networks based on ChIP-seq data. This set of networks allowed us to explore both the impact of including epigenetic data as well as the message-passing optimization in SPIDER. For more information on our data processing and network construction and evaluation pipeline, see Methods.

**Table 1:** Overview of the human cell line data used in this paper.

Cell line	Tissue	Description	# ChIP-seq TFs	# DNase peaks
A549	Lung	Alveolar carcinoma	19	176,870
H1HESC	Stem cell	Male embryo stem cell	35	258,188
HELAS3	Cervix	Cervical adenocarcinoma	38	199,188
HEPG2	Liver	Hepatocellular carcinoma	45	192,959
GM12878	Blood	B-lymphoblastoid cells	58	183,953
K562	Blood	Erythrocytic leukemia	59	202,266

For each cell line we show the number of transcription factors (TFs) with ChIP-seq data and the number of DNase-seq peaks (open chromatin regions).

### SPIDER predicts accurate gene regulatory networks

**Fig. 2:**
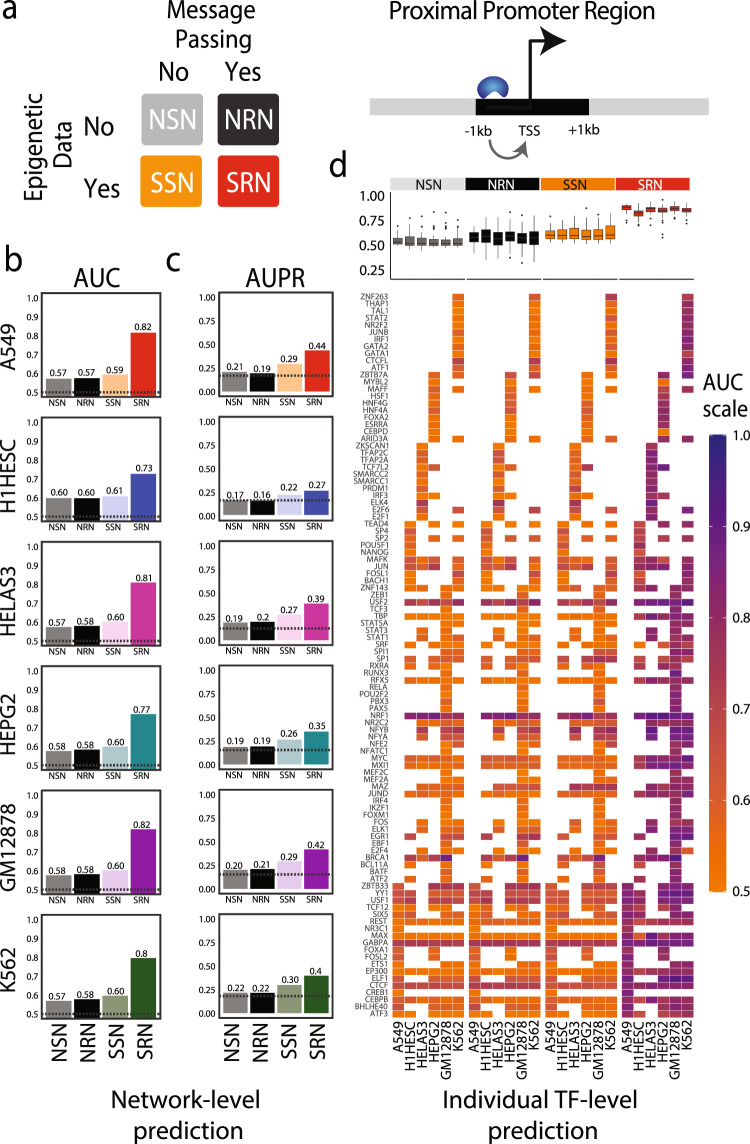
Evaluation of SPIDER predictions. **a** For each cell line we evaluated four networks, modeled either with or without using epigenetic data, as well as with or without applying message passing. SPIDER-predicted regulatory networks represent a combination of epigenetic data and message passing. Throughout the manuscript and figures we refer to these four networks as the naïve seed network (NSN), SPIDER seed network (SSN), naïve regulatory network (NRN), and SPIDER regulatory network (SRN). **b** The AUC values of the four types of networks evaluated in six different cell lines (based on ChIP-seq gold standards). A baseline AUC value of 0.5 is shown as a horizontal dotted line. **c** The AUPR values of the four types of networks evaluated in six different cell lines (based on ChIP-seq gold standards). The baseline AUPR values, equivalent to the percentage of positives in the associated gold standard, are shown as horizontal dotted lines. **d** The AUC values for individual transcription factors (TFs) within each network. The distribution of values is shown in the top panel. Individual values are visualized in the bottom panel. Boxplot elements: center line: median; box limits: 1st and 3rd quartiles; whiskers: upper and lower fence; points: outliers. See also Supplemental File 1, Supplemental Table 1, and Supplemental Figs. 2–4.

To begin, we benchmarked the two naïve networks using the six ChIP-seq “gold standard” networks and evaluated their accuracy based on the Area Under the Receiver-Operating Characteristic Curve (AUC-ROC, or more simply, AUC). This provided a baseline assessment of network accuracy in the absence of epigenetic data (Fig. 2a). Unsurprisingly, we observed very low AUC values – from ~0.57 to ~0.60 (Fig. 2b, gray and black bars)—indicating that message passing in the absence of epigenetic data does not improve the network accuracy. Next, we evaluated the SPIDER seed networks. These networks, by definition, contain a subset of edges from the naïve seed network—those with additional evidence from chromatin accessibility data. We found only a marginal increase in AUC compared to the naïve networks (Fig. 2b, pale colored bars). Initially this result may seem surprising since computational pipelines that use chromatin accessibility information to improve transcription factor binding site prediction often report high accuracy^37,39^. However, it is important to note that we are addressing a different problem. Rather than scoring individual transcription factor binding locations, we are using epigenetic data to prune predicted transcription factor binding sites within gene regulatory regions and define an epigenetically informed network of transcription factor to gene relationships. This recasting of the problem greatly impacts predictive performance compared to what is observed in genome-wide transcription factor binding site assessments (see Supplemental Fig. 1 and Supplemental Section S1).

We next evaluated the SPIDER-predicted gene regulatory networks, which are the result of applying message passing to the epigenetically informed seed networks (Fig. 2b, dark colored bars). We found that SPIDER regulatory networks are highly accurate when benchmarked using the ChIP-seq networks, with AUC scores dramatically increased compared to both the naïve and epigenetically informed SPIDER seed networks. For example, the accuracy of the A549 network was improved by over 37% (AUC = 0.816) compared the seed network. The SPIDER-predicted network for GM12878 was the most accurate, with an AUC value of 0.819. This level of accuracy and overall improvement was consistent across the six cell lines (Supplemental Table 1), demonstrating the robustness of SPIDER in predicting accurate regulatory networks across a range of cell types. Furthermore, high accuracy was not observed when using density-match random networks to seed the message-passing algorithm (see Supplemental Fig. 2 and Supplemental Section S2). Together, these analyses indicate that the combination of epigenetic data and message passing, rather than either in isolation, is critical for identifying an accurate regulatory network.

The ChIP-seq gold standards we used to benchmark our results are slightly unbalanced in terms of class, with an average of 15.6% of the edges in the positive class across the cell lines. Therefore, to ensure that our results were not influenced by issues related to class-imbalance, we repeated these analyses using the Area Under the Precision-Recall Curve (AUPR). We observed almost identical results using AUPR as we did with AUC; across the cell lines, the SPIDER-predicted regulatory networks were always much more accurate than either of the naïve networks or the epigenetically informed seed network (Fig. 2c). We note that, since the baseline for random classification using the AUPR is slightly different for each cell line, the AUC provides a more comparable metric across observations. Since our conclusions are the same using either AUC or AUPR, we opted to use AUC as our primary assessment metric. For reference, AUPR values for all tests are provided in the supplement. The curves associated with the global network AUC and AUPR calculations are provided in Supplemental Fig. 3a.

Next, to ensure that our results were not driven by a handful of transcription factors, such as those with a high number of motif locations or abundant ChIP-seq peaks, we separately evaluated the accuracy of the edges emanating from each individual transcription factor (Fig. 2d, Supplemental Fig. 4a). Just as with the overall networks, we found that the AUC and AUPR values for transcription factors were nearly always significantly higher in SPIDER networks than in the corresponding naïve and seed networks, and that this was true across all the cell lines. Of note, transcription factors with ChIP-seq data in multiple cell lines (CEBPB, CTCF, EP300, GABPA, MAX, REST in all six cell lines; ATF3, USF1, YY1, JUND, MX11, MYC, NRF1, RFX5, TBP, USF2, in five cell lines) had consistent AUC values across the cell lines. Interestingly, some of these transcription factors also have higher AUC values across all the different network types. This may be due to the fact that their corresponding motif is found in the promoter region of more genes. For example, GABPA targets 18–19% of all genes in the seed networks, whereas the average transcription factor only targets about 6% of genes in the seed networks. Other examples include CTCF (targets ~10% of genes), NRF1 (~10%), and USF2 (~7%).

In summary, the predicted SPIDER networks represent the in vivo network structure observed in ChIP-seq data, both at the overall gene regulatory network level and at the level of individual transcription factors. SPIDER’s consistent performance, especially in predicting edges emanating from individual transcription factors (Fig. 2d), indicates that these results are unlikely to be biased by the specific sets of transcription factors used to make each of the ChIP-seq validation networks. Finally, although ChIP-seq evidence for transcription factor binding does not necessarily translate into functional gene regulation^45^, we believe the observed increase in accuracy compared to the naïve and seed networks demonstrates that SPIDER is effectively applying message passing to infer gene regulatory networks.

### SPIDER predicts cell-specific regulatory relationships

A cell’s regulatory network includes interactions that are specific to a given cell type or biological context as well as interactions that are shared across many cell types and support common regulatory processes^3,4^. Therefore, we next evaluated SPIDER’s ability to predict network edges that are cell line specific, i.e., instances where the ChIP-seq data indicates that a transcription factor is bound to the regulatory region of a gene in one cell line but not another. Such interactions are important in determining cellular function and may play a role in a wide range of cell-specific characteristics, including disease risk^46^.

**Fig. 3:**
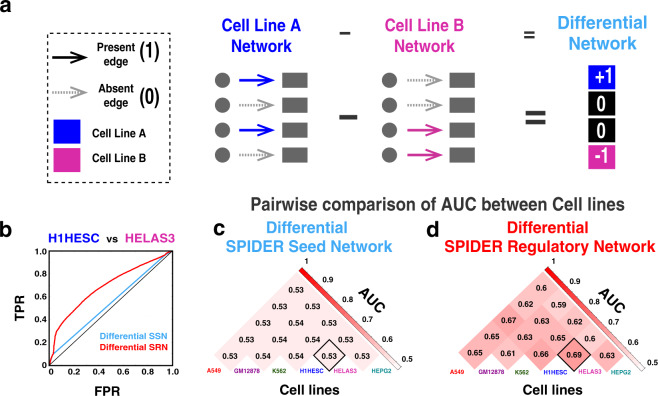
Cell-specific interactions predicted by SPIDER. **a** Illustration of our approach to assessing cell-line-specific network relationships through differential network analysis. **b** ROC curve showing the evaluation of the differential networks that were calculated by comparing the H1HESC and HELAS3 SPIDER seed networks (blue) and regulatory networks (red). **c** AUC values of each of the pairwise comparisons of the SPIDER seed networks. **d** AUC values of each of the pairwise comparisons of the SPIDER regulatory networks.

To evaluate SPIDER’s ability to predict cell-line-specific edges, we first constructed “differential networks”. Specifically, for each pair of cell lines (“A” and “B”), we subtracted (1) the input SPIDER seed networks, (2) the regulatory networks predicted by SPIDER, and (3) the ChIP-seq derived gold standard networks. It’s important to note that, since the seed networks and gold standard networks are binary, taking the difference between these networks results in three classes of edges: those specific to cell line A (difference = +1), those specific to cell line B (difference = −1), and those that are the same in both cell lines (either existing in both, or not existing in both; difference = 0) (Fig. 3a).

Next, we computed the AUC for the differential-seed and differential-regulatory networks by assessing edges that were identified as cell line specific in our differential ChIP-seq networks (see Methods). To help illustrate and interpret this analysis, Fig. 3b shows the ROC curve comparing one pair of cell lines: H1HESC versus HELAS3. For this pair of cell lines, the differential SPIDER regulatory network had a much higher predictive power (AUC = 0.69) compared to the differential-seed network (AUC = 0.53). Importantly, this curve also shows that all the three classes of edges (−1, +1, and 0) in the seed network contribute to the improved AUC. This can be seen by noting that the ROC curve for the differential-seed network is composed of three straight line segments, one for each of the three classes of edges. The ROC for the differential SPIDER network rises above these segments and is especially pronounced for the middle segment. This middle segment represents edges that, simultaneously, either existed or did not exist in both cell lines’ seed networks. Thus, the dramatic shift in the ROC curve for this middle segment indicates that SPIDER is able to identify edges that are cell-line specific (i.e., in only one of the two gold standard networks) even among the portion of the seed network that is identical between the two cell lines. In other words, there are many edges that are the same when comparing the seed networks but that differ in the ChIP-seq gold standards; these edges can be predicted by comparing their weights in the SPIDER networks.

Figure 3c, d illustrate the AUC values for differential networks across all pairs of cell lines. We observe consistently higher values for the differential SPIDER regulatory networks compared to the differential SPIDER seed networks. This indicates that the SPIDER regulatory networks predict cell-line-specific interactions more accurately than the input seed data and suggests that message passing is enhancing the detection of cell-line-specific edges.

### SPIDER can be used to predict distal regulatory elements

Transcription factor binding in the proximal promoter region regulates gene expression through the formation of the pre-initiation complex. Similarly, distal regulatory elements can influence the rate of gene transcription by acting as either activators or repressors^47^. Incorporating these distal regulatory factors into network models is an important step in developing a more holistic perspective on gene regulation.

**Fig. 4:**
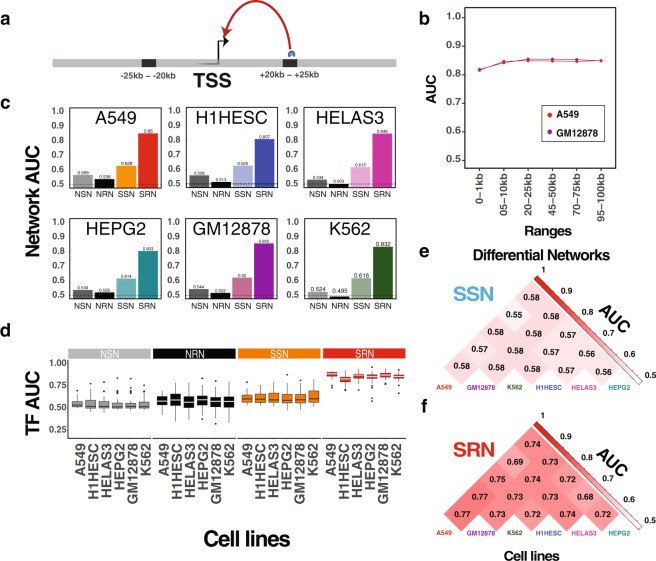
Distal regulatory events predicted by SPIDER. **a** An example set of regions, ±20–25 kb from the transcriptional start site (TSS), used to define distal regulatory windows around a gene. **b** The AUC values for SPIDER predictions across sets of regulatory windows at increasing distance from the transcriptional start site. Two example cell lines (GM12878 and A549) are shown. **c** The AUC values for SPIDER predictions, as well as predictions made without epigenetic data or message passing, for six cell lines using the ±20–25 kb regulatory window. **d** Distribution of AUC values for individual transcription factors. Boxplot elements: center line: median; box limits: 1st and 3rd quartiles; whiskers: upper and lower fence; points: outliers. **e**, **f** Differential analysis demonstrates that SPIDER detects cell-line-specific interactions in distal regulatory windows. As in Fig. 2, NSN Naïve seed network, SSN SPIDER seed network, NRN naïve regulatory network, SRN SPIDER regulatory network. See also Supplemental Table 2, Supplemental Table 3, and Supplemental Figs. 3–4.

One important advantage of chromatin accessibility data such as DNase-seq is the identification of enhancer regions. Although the local chromatin environment around enhancers is well studied^48–52^, less is known about which genes are targeted by these distal elements through mechanisms such as DNA looping^53–55^. However, one simple way to approximate distal regulation is to map predicted transcription factor binding sites to genes using proximity, or the number of base-pairs away a predicted site is from the transcriptional start site of a gene^56^. Along these lines, we modulated the definition of the regulatory region used by SPIDER to assess transcription factor binding sites located outside the proximal promoter. In particular, we defined the regulatory region of each gene as composed of two windows of 5 kb each (total 10 kb) located at various distances upstream and downstream of the transcriptional start site. For example, Fig. 4a shows the regulatory region of a gene as located −20 kb to −25 kb and +20 kb to +25 kb away from the transcriptional start site. For two cell lines, GM12878 and A549, we ran SPIDER using five different definitions of potential regulatory regions: ±5–10 kb, ±20–25 kb, ±45–50 kb, ±70–75 kb, ±95–100 kb. This allowed us to examine the potential impact of distal epigenetic variability on gene regulation at multiple distances; the width of these distal regions was selected such that the density of the SPIDER seed information was similar to our proximal promoter analysis. We benchmarked the results from SPIDER to their corresponding ChIP-seq derived gold standards, also constructed based on these same regulatory regions (Fig. 4b). The AUC values for the regulatory predictions made using these alternate windows showed little variation, indicating that SPIDER can be used to predict transcription factor binding sites outside of the proximal promoter. Interestingly, the prediction accuracy for these distal regulatory elements was even slightly higher than those obtained using proximal promoter region, with an average AUC of 0.85 across distal regions compared to 0.82 for the proximal promoter (Supplemental Table 2).

Given these results, we selected a single window, ±20–25 kb, to investigate in more detail. We systematically evaluated predictions from each of the six cell lines and found that SPIDER accurately predicts distal ChIP-seq binding events. Importantly, the accuracy of SPIDER predictions was significantly (*p* < 2.2 × 10^−16^) higher than either those made based on the epigenetically informed “seed” data or those based on a “naïve” mapping (Supplemental Table 3). This is true both overall (Fig. 4c; Supplemental Fig. 3b) as well as for individual transcription factors (Fig. 4d). As in our previous analysis, we obtained very similar results when computing the AUPR instead of the AUC (Supplemental Fig. 4b, c). We also note that the accuracy of the naïve regulatory predictions was nearly equal to random in the distal analysis (average AUC of 0.52; Supplemental Table 3). This is in contrast to the low, but slightly better than random, predictive performance of the naïve regulatory network constructed for the proximal promoter region (average AUC of 0.58; Supplemental Table 1). This illustrates the importance of incorporating epigenetic data when modeling regulatory connections outside of the proximal promoter and highlights a key strength of the SPIDER approach.

We also evaluated the cell line specificity of these distal regulatory interactions and found that the predictions made by SPIDER were highly cell line specific and more specific than the information used to seed the algorithm (Fig. 4e, f). In addition, SPIDER’s ability to predict cell-line-specific information was higher for distal SPIDER predictions than proximal network predictions (comparing Fig. 4f with Fig. 3d). This is interesting since biological processes specific to individual cell types or tissues are more likely to be driven by distal regulatory elements, such as enhancers, while common “housekeeping” processes tend to be regulated by promoters^57,58^.

### SPIDER outperforms other prediction algorithms

Our results demonstrate that SPIDER can effectively leverage epigenetic data to estimate highly accurate and cell-line-specific regulatory networks. However, there are a number of computational tools that incorporate epigenetic information when performing transcription factor binding site prediction as well as resources that provide networks derived from these types of predictions. We benchmarked the performance of SPIDER using data from several of these resources. In particular, we downloaded publicly available transcription factor binding locations predicted by CENTIPEDE^37^ and PIQ (protein interaction quantitation)^39^, and created a set of associated gene regulatory networks by intersecting these locations with gene regulatory regions using the same pipeline we applied to build the SPIDER seed networks. We also downloaded the networks associated with two publications that analyzed DNase-seq data from ENCODE: Neph et. al.^59^ and Marbach et. al.^60^. Finally, we applied TEPIC^38,61^ to the same DNase-seq data we used to test SPIDER. Additional details regarding how we processed these data is included in Supplemental Section S3.

It is important to note that the data reported by each of these sources varies. For example, it is possible to obtain continuous scores for the predictions made by CENTIPEDE, PIQ, and TEPIC, but the networks provided with the Neph et. al. and Marbach et. al. publications are unweighted. Therefore, to ensure a fair comparison across the sources, we converted all networks (including those estimated by SPIDER) into unweighted graphs using thresholding (see Supplemental Section S3). The cell lines and transcription factors included in each source also differ. Therefore, to gauge the accuracy of network predictions across these sources, we calculated a series of AUC scores by comparing the targeting profile of each transcription factor in a given cell line network with its targeting profile based on cell-line-specific ChIP-seq data. This resulted in a series of AUC scores associated with each method. The number of cell lines, transcription factors, and tests performed for each source is reported in Supplemental Table 4. We note that, by design, the tests performed for each source are always a subset of those performed for SPIDER.

**Fig. 5:**
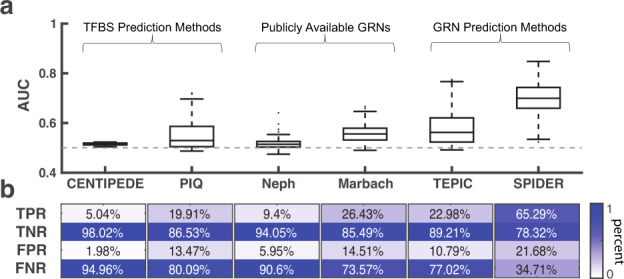
Comparison of SPIDER with other sources. **a** An assessment of the regulatory predictions made by the networks associated with various data sources. The distribution of AUC values across all the tests performed (transcription factor / cell line pairs) is shown. Boxplot elements: center line: median; box limits: 1st and 3rd quartiles; whiskers: upper and lower fence; points: outliers. **b** Heatmap illustrating the median true positive rate (TPR), true negative rate (TNR), false positive rate (FPR), and false negative rate (FNR) across the tests. See also Supplemental Table 4 and Supplemental Figs. 5, 6.

The distribution of the calculated AUC values across all tests is shown in Fig. 5a. We observe that the networks derived from transcription factor binding site prediction algorithms were only marginally better than random chance; the mean AUC across all tests was only 0.516 for CENTIPEDE and 0.556 for PIQ. This is despite the fact that both of these algorithms do an outstanding job of predicting ChIP-seq binding when scoring motif locations (Supplemental Fig. 5a; see also Supplemental Section S1 and Supplemental Fig. 1). The networks reported in Neph et al. and Marbach et al. were also not very accurate, with mean AUCs (based on comparison to ChIP-seq data) across all tests of 0.518 and 0.559, respectively. This is likely due to the fact that these networks were derived using similar techniques as the ones we modeled based on CENTIPEDE and PIQ. The networks predicted by TEPIC were overall more accurate than the other sources (mean AUC = 0.582) but were still less accurate than those predicted by SPIDER (mean AUC = 0.695). We note that these results were not greatly impacted by considering the scores made by the algorithm in lieu of thresholding (Supplemental Fig. 5b) and were similar for distal regulatory interactions (Supplemental Fig. 5d, e). As in the case of our other analyses, these results were also very similar when using AUPR instead of AUC to assess accuracy (Supplemental Fig. 5c, f). Finally, we also point out that SPIDER’s comparative performance is similar when restricting to the subset of tests performed for each source (Supplemental Fig. 6).

To better understand why SPIDER outperformed these other methods/sources, we analyzed the components that lead to the calculation of the AUC (Fig. 5b), namely: (1) the True Positive Rate (TPR), or instances where a transcription factor is predicted to be regulating a gene and that gene has a ChIP-seq peak for the transcription factor in its regulatory region; (2) the True Negative Rate (TNR), or instances where a transcription factor is predicted to be absent and there is no ChIP-seq peak; (3) the False Positive Rate (FPR), or instances where a transcription factor is predicted to be regulating a gene, but that prediction is not supported by ChIP-seq; and (4) the False Negative Rate (FNR), or instances where transcription factor is predicted to be absent, but a ChIP-seq peak exists in the regulatory region of the gene. Visualizing these rates (Fig. 5b) revealed that although other methods generally excel at detecting true negatives, this is at the cost of greatly reducing the number of true positives, ultimately leading to a very high false negative rate and poor overall accuracy. On the other hand, although the networks predicted by SPIDER had a slightly lower true negative rate, they also included many true positive events, which were largely missed by the other methods. This is due to the fact that, unlike previous approaches, SPIDER does not require that a transcription factor motif is present in the regulatory region of a gene in order to predict an interaction between that transcription factor and gene. Rather, an interaction between a transcription factor and a gene can be learned through SPIDER’s message-passing process, which assesses the likelihood of each edge based on the overall structure of the network (see Methods). Biologically, these learned relationships may represent transcription factor regulatory mechanisms that are not captured by DNA sequence^62^, such as the recruitment of cofactors^63,64^.

Finally, we wished to understand how the networks estimated by SPIDER compare to those estimated by methods that leverage transcriptomic data to infer transcription factor to gene connections. Therefore, we identified several commonly used expression-based network reconstruction approaches, including ARACNe^14^, CLR^15^, GENIE3^13^, and PANDA^18^. We applied these approaches to estimate regulatory networks for 666 transcription factors and 19260 genes using lymphoblastoid expression data from the Genotype-Tissue Expression (GTEx) project^3^. We also applied SPIDER to estimate regulatory relationships between these same transcription factors and genes. We then evaluated the accuracy of the networks predicted by each of these algorithms by comparing with the ChIP-seq network for GM12878. We observe that the networks predicted by SPIDER are much more accurate than those predicted by the expression-based network reconstruction methods (Supplemental Section S3 and Supplemental Fig. 7). This analysis illustrates the importance of effectively incorporating epigenetic data into gene regulatory network models, especially for higher-order organisms such as humans.

### SPIDER predicts hidden interactions

In molecular biology, functional validation can often only be performed on a limited number of top predictions. We explored how to use SPIDER’s predictions to develop novel hypotheses regarding transcription factor regulation of genes. Our analysis demonstrates that SPIDER estimates accurate networks by simultaneously predicting two classes of regulatory relationships: those that have initial evidence based on the presence of a transcription factor motif in the regulatory region of a gene, as well as those without evidence from transcription factor motif data but which are instead only supported by the local structure of the regulatory network (and potentially modulated by regulatory mechanisms not encoded in the DNA sequence). We evaluated the potential for generating biologically testable hypotheses regarding novel regulatory interactions using this second class of edges, i.e., edges that were not present in SPIDER’s seed network but had a high edge weight in SPIDER’s predicted regulatory network. For demonstration, we focused on the SPIDER seed and regulatory networks for the A549 (lung cancer) cell line. Key results for each of the six cell lines are included in Supplemental Tables 5-6.

**Fig. 6:**
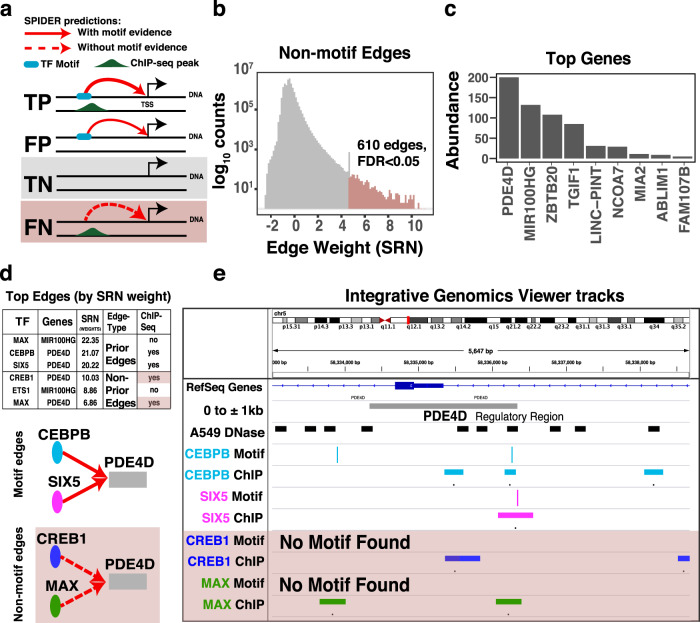
SPIDER Identification of hidden regulatory relationships. **a** Schematic showing the relationship between true positives (TP), false positives (FP), true negatives (TN), and false negatives (FN) in the SPIDER seed network, as well as potential SPIDER predictions (red lines). **b** Distribution of the SPIDER-predicted edge weights in the A549 network for the subset of edges that were absent in the A549 SPIDER seed network (TN or FN edges; see panel **a**). Of these, top-weight edges that have a significant (FDR < 0.05) weight in the SPIDER-predicted networks are shown in light red. **c** The number of times a gene is targeted by one of the top-weight edges shown in panel **b**. **d** A table showing the three top-weight edges predicted by SPIDER that originate from transcription factors with ChIP-seq data. Edges validated by ChIP-seq are illustrated below the table. **e** Integrative Genomics Viewer tracks showing DNase hypersensitivity regions, motif predictions, and ChIP-seq data in the PDE4D promoter region. Motif, DNase, and ChIP-seq peaks exist for CEBPB and SIX5. However, although only DNase and ChIP-seq peaks can be seen for CREB1 and MAX (but no corresponding motif), SPIDER recovered these regulatory relationships. See also Supplemental Table 5 and Supplemental Table 6.

To begin, we selected transcription factor to gene relationships that were absent in the SPIDER seed network (i.e., edges with no evidence of transcription factor regulation of the gene based on intersecting motif data with open chromatin and gene regulatory regions; this set includes both true and false negative edges; Fig. 6a) and plotted the distribution of their weight in the predicted SPIDER regulatory network (Fig. 6b). We then selected the subset of these relationships with the highest weights for further analysis, using FDR < 0.05 as our cutoff (see Methods); these edges are those that were absent in the epigenetically informed seed network but which were subsequently predicted after running SPIDER.

Next, we determined the genes targeted by these edges (Fig. 6c). Among the genes associated with the most edges are several that are important for lung cancer, including *PDE4D* (Phosphodiesterase-4), *ZBTB20*, and *TGIF1*. *PDE4D* is known to promote proliferation and angiogenesis in lung cancer under hypoxia and is a potential therapeutic target for lung cancer therapy^65^. *PDE4D* is also involved in apoptosis, growth, and proliferation in lung cancer cells^66,67^, and promotes the Epithelial-Mesenchymal Transition (EMT) in A549 cell lines^68^. Similarly, *ZBTB20*, a member of the POK family of transcriptional repressors, is upregulated in lung cancer compared to adjacent normal tissue through transcriptional repression of *FOXO1*^69^. Finally, *TGIF1* knockdown inhibits the growth and the migration of non-small cell lung cancer cells^70^ and is dysregulated in several types of cancer. Assessment of *TGIF* expression has shown that silencing *TGIF* attenuates the tumorigenicity of A549 cells^71^. These results suggest that SPIDER networks can identify biologically relevant genes that may be regulated in a context-specific manner despite a lack transcription factor motif evidence.

Finally, we investigated SPIDER-predicted transcriptional regulation, focusing on transcription factors with ChIP-seq data. In particular, we selected the three top-weight edges with motif evidence (true or false positives in the SPIDER seed network) as well as the three top-weight edges that do not have any corresponding motif evidence (true or false negatives in the SPIDER seed network). Four of these top-weight edges, including two with motif evidence and two without, targeted *PDE4D* (Fig. 6d). To better understand these SPIDER predictions, we visualized DNase-seq, ChIP-seq, and motif locations in the regulatory region of *PDE4D* (Fig. 6e). For CEBPB and SIX5 we find ChIP-seq peaks aligned with their corresponding motif and a DNase-seq peak. CREB1 and MAX also have ChIP-seq peaks aligned with DNase-seq peaks but no corresponding predicted motif, meaning that their potential role in regulating *PDE4D* would have been missed using other common approaches. Interestingly, CEBP proteins can mediate the binding of cAMP proteins, such as CREB1, to gene promoters^72^, suggesting that recruitment of CREB1 to *PDE4D* may have been facilitated by CEBPB—a cofactor mechanism which was likely captured by SPIDER’s message-passing procedure.

## Discussion

In this manuscript we present SPIDER, a framework to predict robust, accurate, and epigenetically informed gene regulatory networks. SPIDER works by applying a message-passing approach that emphasizes similarities in transcription factor targeting patterns across an initial (or seed) set of epigenetic-informed regulatory relationships. This process emphasizes consistent structures within the seed network, upweighting edges that originate from transcription factors that have similar sets of target genes as well as edges that point to genes targeted by similar sets of transcription factors. SPIDER not only predicts accurate overall networks, it also reliably estimates cell-line-specific regulatory information. Importantly, SPIDER-predicted networks are significantly more accurate than the regulatory information used to seed the algorithm as well as networks constructed without epigenetic data. This indicates that both message passing and epigenetic data are critical to SPIDER’s success. We recognize that networks derived from ChIP-seq data, like the ones we used to benchmark SPIDER, are not a perfect representation of gene regulation. However, SPIDER’s consistent high performance across multiple cell lines and individual transcription factors gives us confidence both that the algorithm is robustly highlighting true biological signal and that our results are independent of the specific transcription factors that composed our ChIP-seq validation networks.

We also compared the performance of SPIDER-predicted networks to other published gene regulatory networks as well as networks constructed using the results of transcription factor binding site prediction algorithms; all of these networks use similar input data. SPIDER’s predictions were significantly more accurate than those made by other approaches. Other methods often require that a transcription factor’s motif is present in the regulatory region of a gene in order to assign an edge between that transcription factor and gene. Our analysis demonstrates that this leads to a high number of false negatives (missing edges). In contrast, the message-passing procedure employed by SPIDER allows new transcription factor to gene regulatory relationships to be inferred, even when a transcription factor’s motif is not present in the regulatory region of a gene.

SPIDER’s strength lies in its ability to reduce false negatives while retaining a high true positive rate. In other words, SPIDER is able to recover missing edges without introducing a large number of false edges. SPIDER’s ability to detect and effectively enhance hidden interactions not only increases the accuracy of the predicted networks, it also supports the generation of novel hypotheses regarding specific gene regulatory mechanisms. For example, when we investigated edges that had no supporting evidence from our motif scan, but were predicted by SPIDER, we identified CREB1 targeting of *PDE4D* in the A549 network. Not only did we find ChIP-seq evidence of CREB1 binding in *PDE4D*’s promoter region, we also were able to use the network predictions to identify a possible co-factor mechanism mediating this interaction, specifically, the recruitment of CREB1 by CEBPB.

SPIDER is a highly versatile algorithm. In this manuscript, we primarily focused on modeling gene regulatory networks based on promoter regions. However, when we applied the approach to predict transcription factor binding in regions that are distally located from the transcriptional start site, we observed a very high level of accuracy. Like our promoter-based analyses, SPIDER predictions in distal regions were cell-type specific and highly accurate across transcription factors. While we recognize that the ChIP-seq data we used for validation only provides information on transcription factor binding, and not on gene regulation, our ability to accurately predict cell-specific transcription factor binding outside of promoter regions suggests that SPIDER can be used to model distal regulatory mechanisms mediated by enhancers or three-dimensional chromatin structure.

It should also be noted that we only used DNase hypersensitivity as a marker of open chromatin. This was done to facilitate the comparison of SPIDER with existing methods and networks that use DNase-seq data to predict transcription factor binding. However, the algorithm could easily be used with data from other epigenetic marks of open chromatin, such as ATAC-sequencing data or ChIP-sequencing of histones—this is a key future direction of our work. Finally, since SPIDER builds on the message-passing framework used in the PANDA reconstruction algorithm, it has the potential to be extended to incorporate other sources of regulatory information, including protein–protein interaction and gene expression data (see Supplemental Section S4 and Supplemental Fig. 8).

SPIDER provides a principled way to use open chromatin data to gain a comprehensive understanding of the cellular transcriptional regulatory architecture. The algorithm’s unique application of message passing to highlight structures in a seed network gives it a distinct advantage compared to other methods and illustrates the importance of considering the overall regulatory context when predicting transcription factor targeting. SPIDER predicts biologically interpretable, context-specific, and epigenetically informed gene regulatory networks. Ultimately, we believe SPIDER networks will facilitate a more comprehensive understanding of regulatory processes that define health and disease.

## Methods

### Details of the SPIDER algorithm

SPIDER combines (1) a simple approach for identifying transcription factor binding sites in open chromatin, with (2) a network reconstruction algorithm that can overcome issues related to missing information, such as the potential for a transcription factor to bind to DNA without a corresponding sequence motif. SPIDER consists of four main steps.

#### Step 1—Intersect open chromatin regions with motif locations

First, SPIDER uses bedtools^73^ (version v2.25.0) to intersect a BED file containing regions of open chromatin with a series of BED files containing the locations of transcription factor motifs (one BED file per transcription factor). The output of this step is a single BED file that contains the locations of transcription factor motifs that are in open chromatin regions. By default, each of these locations is given a score of one. Note that, in practice, the file produced by this step could be produced in another manner and still used by SPIDER.

#### Step 2—Intersect motifs in open chromatin (from Step 1) with gene regulatory regions and create a seed regulatory network

Next, SPIDER uses bedtools to overlap a BED file containing the locations of transcription factors that are in open chromatin (created in Step 1) with a BED file containing the regulatory regions of genes; a gene can have multiple associated regulatory regions in this second file. If a transcription factor’s motif falls within the regulatory region(s) of a gene, then an edge is created between that transcription factor and gene. The maximum score across all transcription factor motif instances associated with a gene is used to weight the edge; by default, this value is one. The result of this step is an epigenetically informed seed regulatory network between all transcription factors and genes.

#### Step 3—Degree normalize seed network

The seed network from Step 2 consists of edges between *N*_TF_ transcription factors and *N*_G_ genes. In this network, edges connected to high degree nodes, especially high degree genes, also tend to be associated with many open chromatin regions and are more likely to be biologically relevant. Let us denote the transcription factor by gene adjacency matrix describing the seed network as *A*. Based on this matrix, we can calculate the average degree for each transcription factor *i* and gene *j* as:

1 $$k_i^{{{{\mathrm{TF}}}}} = \frac{1}{{N_{{{\mathrm{G}}}}}}\mathop {\sum}\nolimits_j^{N_{{{\mathrm{G}}}}} {A_{ij}} \quad {{{\mathrm{and}}}}\quad k_j^{{{{\mathrm{Gene}}}}} = \frac{1}{{N_{{{\mathrm{TF}}}}}}\mathop {\sum}\nolimits_i^{N_{{{\mathrm{TF}}}}} {A_{ij}} .$$

We use this information to degree normalize the seed network:

2 $$A_{ij}^ \ast = A_{ij}\sqrt {(k_i^{{{{\mathrm{TF}}}}})^2 + (k_j^{{{{\mathrm{Gene}}}}})^2} .$$

#### Step 4—Apply message passing

Finally, SPIDER applies the PANDA^18^ message-passing algorithm to the degree-normalized seed network A* calculated in Step 3. PANDA’s message-passing framework integrates information from three networks, representing transcription factor protein–protein interactions (*P*), transcription factor to gene regulatory interactions (*W*) and gene co-expression (*C*) (see “Details of the PANDA algorithm” below). In SPIDER, *P* and *C* are set equal to the identity matrix; *W* is set equal to A*. PANDA returns a complete, bipartite network with edge weights representing the likelihood that a transcription factor regulates a gene; the distribution of these edge weights is similar to Z-scores.

#### Summary

In step 1, SPIDER identifies potential transcription factor binding sites within open chromatin regions. Step 2 transforms those predictions into a bipartite network describing relationships between transcription factors and genes. Step 3 adjusts the weights of edges according to biological knowledge and in a manner that counter-balances the Z-score transformation applied by PANDA to the input data matrices (see “Details of the PANDA algorithm” below). Finally, in step 4, SPIDER applies the PANDA network reconstruction approach to the degree-adjusted seed network.

### Details of the PANDA algorithm

SPIDER repurposes the message-passing approach implemented in PANDA^18^. While PANDA has been extremely successful, it does not incorporate epigenetic data. In other words, the input seed network used by PANDA generally assumes that all motif sites on the genome are equally accessible.

Supplemental Figure 9 provides an overview of PANDA’s message-passing procedure. PANDA harmonizes data contained in input three matrices: (1) *W* is a *N*_TF_ by *N*_G_ matrix that represents an initial approximation of transcription factor to gene regulatory relationships; (2) *P* is a *N*_TF_ by *N*_TF_ matrix that represents an initial approximation of cooperative interactions between transcription factor proteins; and (3) *C* is a *N*_G_ by *N*_G_ matrix that represents an initial estimate of whether pairs of genes are co-regulated. Since diverse data is often used to construct these matrices, they are immediately normalized by transforming the matrix values into Z-scores using:

3 $$X_{ij}^{(0)} = \frac{1}{{\sqrt 2 }}\left( {\frac{{X_{ij} - \mu _i}}{{\sigma _i}} + \frac{{X_{ij} - \mu _j}}{{\sigma _j}}} \right),$$

where *μ*_*i*_/*σ*_*i*_ and *μ*_*j*_/*σ*_*j*_ are the mean/standard-deviation across row *i* and column *j* of *X*, respectively; *X* can be either *W*, *P*, or *C*. The data across these three normalized matrices is then iteratively updated through a series of normalized matrix multiplications using:

4 $${{{\mathcal{T}}}}_{ij} = {{{\mathcal{T}}}}({{{\vec{\boldsymbol x}}}},{{{\vec{\boldsymbol y}}}}) = \frac{{{{{\vec{\boldsymbol x}}}} \cdot {{{\vec{\boldsymbol y}}}}}}{{\sqrt {\left\| {{{{\vec{\boldsymbol x}}}}} \right\|^2 + \left\| {{{{\vec{\boldsymbol y}}}}} \right\|^2 - \left| {{{{\vec{\boldsymbol x}}}} \cdot {{{\vec{\boldsymbol y}}}}} \right|} }},$$

where ${{{\vec{\boldsymbol x}}}}$ and ${{{\vec{\boldsymbol y}}}}$ are vectors representing row *i* of matrix *X* and column *j* of matrix *Y*, respectively. At each message-passing step, *t*, *W* is updated using:

5 $$W^{(t)} = \left( {1 - \alpha } \right)W^{(t - 1)} + \frac{\alpha }{2}\left( {{{{\mathcal{T}}}}(P^{(t - 1)},W^{(t - 1)}) + {}^{{{\mathrm{T}}}}{{{\mathcal{T}}}}(C^{(t - 1)},{}^{{{\mathrm{T}}}}W^{(t - 1)})} \right)$$

*P* and *C* are then updated using

6 $$\begin{array}{l}P^{(t)} = \left( {1 - \alpha } \right)P^{(t - 1)} + \alpha {{{\mathcal{T}}}}\left( {W^{(t)},\,{\,}^{{{\mathrm{T}}}}W^{(t)}} \right)\, {{{\mathrm{and}}}}\\ C^{(t)} = \left( {1 - \alpha } \right)C^{(t - 1)} + \alpha {{{\mathcal{T}}}}\left( {{\,}^{{{\mathrm{T}}}}W^{(t)},\,W^{(t)}} \right).\end{array}$$

The update parameter, *α*, can take a value between 0 and 1, but by default is set equal to 0.1. ^T^*W*^(*t*)^ indicates transpose of *W*^(*t*)^. These updates are repeated until algorithm convergence.

We note that in SPIDER, we set both *P* and *C* equal to the identity matrix (see “Details of the SPIDER algorithm” above). In this case, *W*^*(1)*^*~W*^*(0)*^, *P*^*(1)*^ can be interpreted as a normalized quantification of the number of shared gene targets between pairs of transcription factors in *W*^*(1)*^, and *C*^*(1)*^ can be interpreted as a normalized quantification of the number of shared transcription factor regulators between pairs of genes in *W*^*(1)*^_._ The off-diagonal elements in *P*^*(1)*^ and *C*^*(1)*^ are then propagated back into *W*^*(t>0)*^ through the subsequent message-passing steps. Thus, this message-passing procedure can leverage the topological structure of the seed network defined by *W*^*(0)*^ to identify transcription factor to gene relationships that are absent in the seed network.

In this paper we develop SPIDER using the MATLAB implementation of PANDA due to its speed and readability^74^. However, PANDA is also implemented in C, python, and R, supporting future implementations of SPIDER in these languages (https://netzoo.github.io/).

### Data used to test SPIDER

The input data used by SPIDER include BED files which contain (1) the genomic locations of potential transcription factor binding sites (one BED file per transcription factor), (2) epigenetic (chromatin accessibility) information, and (3) regulatory regions. We used hg19 coordinates for all the data in this study.

#### Identification of potential transcription factor binding sites

We identified motifs, in the form of position weight matrices, for 687 human transcription factors from the Catalog of Inferred Sequence Binding Preferences (Cis-BP)^43^ (http://cisbp.ccbr.utoronto.ca, accessed: July 7, 2015). We mapped the motifs to the human genome (hg19) using FIMO^42^ with a custom background model for promoter sequences, and retained all locations meeting a significance of *p* < 10^−4^.

#### Epigenetic data

We obtained DNase-seq peak locations from ENCODE for six cell lines. Additional information about the DNase-seq data used, including lab and download URL, is given in Supplemental File 1.

#### Regulatory regions

We used RefSeq gene annotations downloaded from the UCSC genome browser (https://genome.ucsc.edu/cgi-bin/hgTables; accessed on 29 May 2018) to define the regulatory regions of 27,090 genes. We defined regulatory regions in terms of distance from the transcription start site: the proximal regions were defined as a window of 2 kb centered around the transcriptional start site. For the analysis where we evaluated the potential of SPIDER to be used to predict distal regulatory events, we used a pair of ranges, each with a width of 5 kb. We chose separate distal windows of 5 kb on both sides of the transcriptional start site in order to exclude promoter effects and to keep the density of regulatory seed network information similar between the promoter and distal analyses. For our primary distal analysis, these ranges were situated at a distance of 20–25 kb both upstream and downstream of the transcriptional start site. We also evaluated SPIDER at various distance-ranges in the GM12878 and A549 cell lines. This included windows at ±5–10 kb, ±20–25 kb, ±45–50 kb, ±70–75 kb, and ±95–100 kb around the transcriptional start site. We saw little variation in the accuracy of SPIDER predictions across these ranges.

### Data used to validate SPIDER

We obtained ChIP-seq data for human transcription factors in six cell lines from ENCODE in narrow peak (BED) format. Information about the ChIP-seq data used, such as treatment, antibody, data freeze date, lab, and download URL, is provided in Supplemental File 1. For some transcription factors, multiple ChIP-seq experiments performed in the same cell line were available. In these cases, to reduce the potential for false negatives in our benchmark^75,76^, we made one composite file containing all ChIP-seq peaks using the bedtools merge function. To create gold standard networks from these data, ChIP-seq peaks were intersected with gene regulatory regions following the same protocol as used by SPIDER, Step 2, as described above.

When benchmarking, we took the intersection of the transcription factors in the predicted network and those in the corresponding ChIP-seq network, and evaluated the subnetwork corresponding to all possible edges between the overlapping transcription factors and all target genes. For example, for A549 this corresponded to a subnetwork containing the 514,710 edges between 19 transcription factors and 27,090 target genes (Table 1). We computed AUC and AUPR values using the *perfcurve()* function in matlab (R2014b). To calculate AUPR for the seed networks (which are binary), we added a small level of Gaussian random noise (sigma = 0.05) to the initial 0/1 edge weights. For the differential network analysis, we analyzed the subnetwork associated with transcription factors assayed in both cell lines and used *perfcurve()* with the ‘negclass’ parameter to assign both a positive (+1) and negative (−1) prediction category; this excluded evaluation of edges that were identical in both cell line ChIP-seq networks (0).

### Detecting hidden interactions inferred by SPIDER

For each cell line network, we selected transcription factor to gene relationships that were absent in the SPIDER seed network. Because SPIDER uses PANDA to perform message passing, the weights of these edges can be interpreted as Z-scores^18^ (see “Details of the PANDA algorithm” above). Therefore, to identify significant edges in this class, we converted the weights of edges into probabilities using the *pnorm()* function in R and corrected for multiple comparisons using the Benjamini–Hochberg method. We selected top significant edges for further analysis using an FDR cutoff of 0.05. For the A549 proximal network this corresponded to an edge weight cutoff of 4.64.

## Supplementary information

Supplementary Information

Supplementary Data 1

## Data Availability

A complete list of all ENCODE data files used in our analysis, along with their locations on the ENCODE DCC website, is contained in Supplemental File 1.

## Code availability

An implementation of SPIDER is available at: https://github.com/kimberlyglass/spider. The input data and output networks analyzed in this manuscript can also be directly downloaded from: https://sites.google.com/a/channing.harvard.edu/kimberlyglass/tools/spider.
