# Supplementary material for: Constructing gene regulatory networks using epigenetic data
Source: NPJ Syst Biol Appl. 2021 Dec 9;7:45. doi: 10.1038/s41540-021-00208-3 (PMC8660777; doi:10.1038/s41540-021-00208-3)
Supplement: Supplementary file 1 — Supplementary Information [file 41540_2021_208_MOESM1_ESM.pdf]

Supplementary Materials and Methods

S1. Limitations to using TFBS predictions when constructing gene regulatory networks

Position-weight-matrices (PWMs) are used to conceptualize the DNA sequence pattern bound by a transcription factor (TF). The genomic locations ("motif locations") that match the pattern contained in a PWM can be used to predict a transcription factor's binding sites (TFBSs). Multiple methods have been developed that leverage chromatin accessibility and other genomic information (e.g. sequence conservation, proximity to an annotated transcription start site, etc.) alongside PWMs to improve motif scoring and thereby TFBS predictions. These methods are generally benchmarked by comparing the scores associated with each motif location against a "gold standard" derived from ChIP-seq data. At a technical level, this amounts to comparing two vectors of length N_m, where N_m is the number of motif locations in the genome: (1) one containing scores associated with each motif location, and (2) one indicating whether or not there is also a ChIP-seq peak at these locations. This approach has two well-known limitations. First, by definition, this approach excludes locations in the genome where the TF is bound based on ChIP-seq data but there is no corresponding pattern in the DNA that matches the PWM. Secondly, because only a handful of motif locations are occupied by a transcription factor, these evaluations are prone to a high number of true-negatives, which can artificially inflate metrics such as the Area Under the Receiver-Operating Characteristic Curve (AUC-ROC, or more simply AUC).

These issues, especially the former, are exacerbated when constructing gene regulatory networks. In constructing gene regulatory networks, the motif locations that occur within the regulatory region (most often the promoter region) of a gene are often used to estimate which TFs regulate that gene. In contrast to genome-wide TFBS prediction, benchmarking the targets of a TF in a gene regulatory network involves comparing two vectors of length N_g, where N_g is the number of genes in the network: (1) one containing information regarding whether or not the TF's motif was found within the regulatory region(s) of the genes, and (2) one containing information regarding whether or not there is a ChIP-seq peak for that same TF within the regulatory region(s) of the genes. Note that in this evaluation, genes whose regulatory regions contain a bound TF based on ChIP-seq data, but do not contain a corresponding match to that TF's PWM, are no longer excluded, but instead are considered false negatives.

This shift has profound implications for model accuracy. Namely, even highly accurate TFBS predictions can, when naively used to construct a gene regulatory network as described above, lead to very poor network accuracy. To illustrate this seemingly counter-intuitive statement, **Supplemental Figure 1** depicts a toy example illustrating how the same data evaluated using either a "motif-centric" or a "gene-centric" approach can lead to dramatically different AUC values. The top panel shows three tracks that represent the genomic locations of (1) motifs, (2) gene promoters, and (3) open chromatin (for example, DNase-seq peaks). The middle panel shows the location of TF binding sites based on ChIP-seq peaks. In constructing this example, we selected parameters consistent with those observed in real biological data. Namely, 75% of gene promoters also contained an open chromatin region and 40% contained a predicted TFBS, 5% of predicted TFBS not in promoters were in regions of open chromatin (representing enhancers), and 25% of open chromatin regions contained a true TF binding site (ChIP-seq peak); no ChIP-seq peaks were located outside of open chromatin regions.

We computed the AUC using both a motif-centric and gene-centric approach. For the motif-centric comparison, motif locations that overlapped with open chromatin were given a score of "1" while motif locations that were not in open chromatin were given a score of "0". Benchmarking this vector of motif scores against the ChIP-seq peaks resulted in a very high AUC value (AUC = 0.96; **Supplemental Figure 1**, first row of bottom panel). For the gene-centric approach we considered two scenarios. In the first, gene promoters that contained a motif were give a score of "1" and promoters that did not contain a motif were given a score of "0". This is equivalent to using motif locations without chromatin information to predict a TF's target genes, as in a regulatory network. In this set-up, the AUC value dropped severely (AUC = 0.55; **Supplemental Figure 1**, second row of bottom panel). For the second scenario, gene promoters that contained a motif that was also in open chromatin were given a score of "1" and all other gene promoters were given a score of "0". In this case, despite the additional epigenetic information, there was only a minimal improvement in AUC (AUC = 0.63; **Supplemental Figure 1**, third row of bottom panel), and the accuracy was significantly below that obtained using the same information, but in a motif-centric manner.

This analysis shows that highly accurate results from motif-centric prediction approaches, including algorithms that combine PWM and chromatin accessibility information to predict TFBS, do not directly translate into accurate gene regulatory network models. This is not only due to a high number of true negatives, but also a high number of false negatives.

S2. Evaluating SPIDER's performance when using random-like seed networks

At some level, a naïve network that is constructed by intersecting the locations of transcription factor binding sites and gene regulatory regions, can be thought of as "random-like" since it does not include any cell-type specific information. An additional intersection with open chromatin regions essentially prunes edges from this naïve network by incorporating information regarding the specific biological context measured in the associated experimental assay, such as cell type or exposure condition. One of the assumptions of SPIDER is that this pruned network contains biologically relevant patterns that can be identified and amplified by message-passing in order to improve accuracy.

We investigated this assumption by evaluating whether pruning a naïve network using DNase hypersensitivity data is equivalent to creating a lower-density "random-like" network that can be improved using message-passing, or if epigenetically informed pruning results in a non-random network, i.e. one that includes patterns that can be identified and amplified by message-passing in order to improve accuracy. To do this, we began with the naïve seed network analyzed in the main text. Then, for each cell line, instead of using DNase hypersensitivity data to create a SPIDER seed network, we randomly pruned edges from the naïve seed network to create a random-like seed network that had the same number of edges as the SPIDER seed network. We next applied message-passing to each of these random-like seed networks to create corresponding random-like regulatory networks. We repeated this ten times. **Supplemental Figure 2** shows the AUC and AUPR values for the naïve seed, SPIDER seed, and SPIDER regulatory network in each cell line, as reported in **Figure 2**, as well as the mean AUC and AUPR values across the ten random-like seeds for each cell line and across the ten corresponding random-like regulatory networks for those cell lines.

First, we see that the random-like seed networks are much less accurate than the naïve seed network (comparing the pale purple and gray bars). This is because the naïve seed network is not random but contains biological signal derived from the existence of transcription factor binding sites in the regulatory regions of genes. Thus, randomly pruning edges is equivalent to adding noise to the biological signal in the naïve seed network, resulting in lower accuracy. We also see that the message-passing procedure marginally improves network accuracy when applied to the random-like seeds (comparing dark purple and light purple bars). We believe that this is due to biological signal from the naïve seed network that is retained despite the random pruning. Consistent with this, these random-like regulatory networks (at best) approach the accuracy of the naïve seed network. In contrast, as reported in the main text, the epigenetically informed SPIDER seed networks have a slightly higher accuracy than the naïve seed network (comparing the orange and gray bars) and applying message-passing to the epigenetically informed SPIDER seed networks results in highly accurate predictions (red bars).

Overall, this analysis illustrates that the employed seed networks are not random-like. In fact, the naïve seed network contains biological signal that is degraded upon random pruning, but that is slightly improved when pruning based on epigenetic data (as is done with SPIDER). We believe the message-passing procedure is identifying this signal and using it to further improve network accuracy.

S3. Benchmarking SPIDER against other methods

We compared the performance of SPIDER to that of several other methods and available networks. The methods and networks that incorporate epigenetic information fall into three main categories: (1) transcription factor binding site prediction methods (whose output needs to be processed to create gene regulatory networks comparable to those predicted by SPIDER); (2) publicly available gene regulatory networks (provided as a resource in other publications), and (3) gene regulatory network prediction methods. We also compared SPIDER to (4) algorithms that are designed to estimate a network using gene expression data. In analyzing these diverse

methods and resources, we processed the provided data in as consistent of manner as possible to support a fair comparison of the sources.

Below we describe each of these additional methods/networks and how we adapted their usage or output to systematically compare their performance with each other and with that of SPIDER. For all methods/networks we only focused on the subset of cell lines and transcription factors for which we had ChIP-seq data (see **Supplemental File 1**). This means that the evaluations we perform for each method/network are a subset of the evaluations we perform for SPIDER. For each of the methods/networks we performed a threshold analysis to evaluate the accuracy of that method/network's predictions. The thresholds used in these analyses were used to calculate the true positive rate, true negative rate, false positive rate, and false negative rate for each test performed, which is summarized in **Figure 5b** in the main text. The results reviewed in this section are summarized in **Supplemental Table 4** and illustrated in **Figure 5a** and **Supplemental Figures 5-6**.

Group 1: TFBS inference methods

We chose CENTIPEDE[1] and PIQ[2] as two representative, highly-cited examples of the many methods that have been developed to perform genome-wide TFBS prediction. Both of these were pioneering methods and are widely used as benchmarks.

<u>CENTIPEDE</u>: The CENTIPEDE algorithm identifies regions of the genome that are bound by transcription factors using a hierarchical Bayesian mixture model to integrate histone modifications and DNase I cleavage patterns with gene annotations and evolutionary conservation. We downloaded the data on TFBS predicted by CENTIPEDE from two resources:

(a) the original CENTIPEDE repository: http://centipede.uchicago.edu/SimpleMulti/
(b) website hosting supplemental material for [3]: https://noble.gs.washington.edu/proj/priors4search/

From the original CENTIPEDE repository, we downloaded the TFBS predictions for five cell lines (GM12878, H1HESC, HELAS3, HEPG2, K562) since these were analyzed in our primary SPIDER analysis. These data listed the genomic locations of predicted TFBS in hg18 coordinates, which we converted to hg19 coordinates using the liftOver program (downloaded from https://genome.ucsc.edu/cgi-bin/hgLiftOver, accessed October 2, 2018), but no scoring information. Therefore, to provide additional insight into the performance of CENTIPEDE, we also downloaded information regarding the posterior probability of TF binding data for the CENTIPEDE model, which was provided as part of the supplemental material in [3]. These data included six TFs in one exemplar cell line: GM12878. Although limited, these data included scores for all motif locations, allowing us to recapitulate the performance of CENTIPEDE at a genome-wide level and to gain insights into why the apparent superior performance of the algorithm did not directly translate into accurate network predictions (see **Section S1**).

To evaluate CENTIPEDE, we first focused on the limited dataset from [3] and calculated the accuracy of the predicted motif locations based on PWM scores as well as the CENTIPEDE-predicted posterior probability scores. In particular, for each TF, we intersected the associated motif locations with the locations of ChIP-seq peaks (using the same ChIP-seq data as we used to benchmark SPIDER); locations that intersected with a ChIP-seq peak were considered true binding events. When then calculated the AUC based on the initial PWM scores associated with the motif locations as well as using the posterior probability scores predicted by CENTIPEDE. This analysis recapitulated CENTIPEDE's excellent predictive performance, with a mean AUC of 0.9782 across the six TFs based on the posterior probability score. This compared to a mean AUC of only 0.7039 when using the original PWM scores (**Supplemental Figure 5a**).

Next, for each TF, we intersected motif locations with either proximal (±1kb around the TSS) or distal (±20-25kb around the TSS) gene regulatory regions (as in Step 2 of SPIDER). Genes for which no associated regulatory region included a motif location were given a default weight of 0.001 less than the minimum posterior probability score across all motif locations, genes for which exactly one motif location was found across all associated regions were assigned the posterior probability score associated with that motif location, and genes for which more than one motif location was identified across all associated regulatory regions were assigned the highest of the posterior probability scores associated with the identified motif locations. For each TF, we compared the resulting vector of gene scores with information regarding whether or not there is also a ChIP-seq peak for that TF within at least one of the gene's associated regulatory regions; we then used this information to calculate

both an AUC and AUPR for that TF. We found that the mean AUC across the six TFs was only 0.5403 for the proximal regions (**Supplemental Figure 5b-c**) and 0.5632 for the distal regions (**Supplemental Figure 5e-f**).

We also performed a threshold analysis since this would better align with the data supplied in the original CENTIPEDE repository. In particular, we identified motif locations that had a high posterior probability (score > 5) and intersected those locations with proximal and distal gene regulatory regions (as in Step 2 of SPIDER). Genes which were not associated with any regulatory regions that included one of these high-scoring motif locations were given a default weight of zero, while genes associated with at least one regulatory region that contained one or more of these high-scoring motif locations were given a score of one. The mean AUC across the six TFs in this context was only 0.5236 for the proximal regions and 0.5189 for the distal regions (**Supplemental Table 4**).

Finally, we analyzed the data provided from original CENTIPEDE repository, which contained bed files listing only high-probability motif locations. Exactly as in the above analysis, we intersected motif locations with gene regulatory regions. We merged predictions from multiple motifs that mapped to the same TF and then benchmarked each resulting profile with its corresponding standard based on ChIP-seq data for that TF and cell-line (47 total comparisons). We observed overall poor predictive performance, with a mean AUC of only 0.5156 for proximal regions and 0.5218 for distal regions. These values are very similar to those we obtained when analyzing the more detailed data from only six TFs, as described above. The results of these analyses are shown in **Figure 5a** (proximal) and **Supplemental Figure 5d** (distal).

Protein-DNA Interaction quantitation (PIQ): PIQ is a tool for global TF binding site detection using a combination of motif information and DNase-seq data[2]. Similar to CENTIPEDE, PIQ estimates binding probabilities associated with a set of motif locations. We downloaded data containing PIQ predictions from http://piq.csail.mit.edu/data/141105-3618f89-hg19k562.calls/. These data contained predictions for the K562 cell line and included 48 motifs that were also associated with one of the TFs for which we had K562 ChIP-seq data; these 48 motifs corresponding to 25 unique TFs.

As in our CENTIPEDE analysis (see above), we first evaluated the overall accuracy of the PIQ scores across all motif locations. More specifically, for each motif, we merged together the genomic locations provided for both the standard and reverse compliment PIQ analyses. We then intersected these combined locations with the locations of ChIP-seq peaks (using the same ChIP-seq data as we used to benchmark SPIDER); locations that intersected with a ChIP-seq peak were considered true binding events. We used these data to calculate an AUC based on the initial PWM scores as well as based the scores assigned by PIQ. In this context, PIQ performed well, with a mean AUC of 0.7729 across the 48 motifs based on the PIQ score, compared to a mean AUC of 0.6001 for the PWM score (**Supplemental Figure 5a**).

Next, we intersected motif locations with either proximal or distal regulatory regions (as described in Step 2 of SPIDER). Exactly as in our CENTIPEDE analysis (see above), we merged predictions from multiple motifs that mapped to the same TF, genes whose regulatory regions didn't include a motif location were given a default weight of 0.001 less than the minimum score across all motif locations, genes associated with exactly one motif location across all associated regulatory regions were assigned the score associated with that motif location, and genes with more than one motif location identified across all associated regulatory regions were assigned the highest score across all associated motif locations. For each motif, we compared the resulting vector of gene scores with information regarding whether or not there is also a ChIP-seq peak for the corresponding TF within at least one of the gene's associated regulatory regions; this allowed us to calculate an AUC and AUPR for that motif. We found that the mean AUC across the 48 motifs was only 0.5703 for the proximal regions (**Supplemental Figure 5b-c**) and 0.5606 for the distal regions (**Supplemental Figure 5e-f**).

Finally, we also performed a threshold analysis where we identified motif locations that had a high score (score > 0) and intersected those locations with proximal and distal gene regulatory regions (as in Step 2 of SPIDER). Genes whose associated regulatory regions didn't include a high-scoring motif location were given a default value of zero, while genes that were associated with at least one regulatory region that contained one or more high-scoring motif locations were given a score of one. The mean AUC across the 48 tested motifs was 0.5559 for proximal regions and 0.5389 for distal regions. The results of these analyses are shown in **Figure 5a** (proximal) and **Supplemental Figure 5d** (distal).

Overall, these analyses indicate that the predictions from CENTIPEDE and PIQ cannot be naively used for regulatory network inference. TFBS prediction approaches that combine motif locations with chromatin data are poor predictors of TF occupancy when restricted to gene regulatory regions. For more information, see **Section S1**, above.

Group 2: Available gene regulatory networks

Networks published along with Neph et. al.[4]: One of the first attempts to use DNase footprints to construct regulatory networks was published by Neph et. al.[4]. In this paper, the authors combined TF motif locations with genome-wide DNase1 footprints to construct regulatory networks of transcription factors. Specifically, if the DNase1 footprint of transcription factor *i* was identified within the promoter region of the gene encoding transcription factor *j*, a regulatory edge was drawn from transcription factor *i* to transcription factor *j*. By design, only genes that encode transcription factors were included in these networks.

We downloaded the regulatory networks associated with [4] from http://www.regulatorynetworks.org. From these, we selected the networks for the K562 and HEPG2 cell lines, since those were included in the six cell lines for which we had ChIP-seq data. Note that the downloaded networks were binary (non-weighted), only contained transcription factors, and were restricted to promoter-based regulatory predictions. We evaluated the accuracy of each of the TFs within these networks using the ChIP-seq gold standards used to evaluate TFs in the proximal SPIDER networks, restricting those standards to only include target genes within in the Neph et. al. networks (i.e. the subset of genes encoding transcription factors). The mean AUC across all the tests performed was 0.5178, similar to mean AUC for threshold proximal CENTIPEDE analysis. The results of this analysis are illustrated in **Figure 5a** in the main text.

Networks published along with Marbach et. al.[5]: Another collection of gene regulatory network models was provided along with the article published by Marbach et. al.[5]. These network models were constructed by identifying the promoters and enhancers of genes using CAGE expression profiles, and then linking TF motif locations found within these regions to the corresponding target genes.

We downloaded the networks constructed by Marbach et. al.[5] from http://regulatorycircuits.org. Among the 394 networks provided through this resource, we identified five for which we had corresponding ChIP-seq data and could use for benchmarking and validation. The downloaded networks were binary (non-weighted), limited to the set of genes used for network building in the CAGE analysis (these genes were provided by the authors in a supplemental file together with the downloaded networks), and did not distinguish between proximal and distal regulation. Similar to how we evaluated the networks associated with Neph et. al. (see above), we evaluated the accuracy of these networks using the ChIP-seq gold standards used to evaluate the proximal SPIDER networks. Specifically, we restricted each gold standard to only include the set of genes used in the CAGE analysis and evaluated each TF in each network using its corresponding TF and cell line ChIP-seq standard (167 total tests). The mean AUC across all the tests performed was 0.5595, similar to the mean AUC for the threshold proximal PIQ analysis. The results of this analysis are illustrated in **Figure 5a** in the main text.

Group 3: Gene regulatory network prediction methods

TEPIC[6, 7]: TEPIC performs a segmentation-based method to predict TF-binding by the combining PWMs and open-chromatin assay data. TEPIC computes TF binding affinities based on a biophysical model of TF binding which detects low affinity binding sites. Of all the methods/networks evaluated, the TEPIC input information and approach is the most similar to that of SPIDER.

Reconstructing networks using TEPIC: We downloaded the TEPIC program and supporting files from https://github.com/SchulzLab/TEPIC. To create regulatory predictions, the input to TEPIC includes (1) a set of formatted PWMs, (2) the full genomic sequence in FASTA format, (3) open chromatin regions in BED format, (4) a gene annotation file that indicates the location of gene transcriptional start sites, and (5) a window size to indicate the range around the TSS to designate as the regulatory region. For (1) we used a set of formatted JASPAR motifs ("human_jaspar_hoc_kellis.PSEM") provided with the TEPIC program. For (2) we used the same

hg19 fasta sequence file used for the FIMO motif scan we performed to create the SPIDER input files. For (3) we used the same DNase peak bed files we used when running SPIDER.

For (4) we created custom gene annotation files that would ensure TEPIC used the same regulatory regions that we used when running SPIDER. In particular, for the proximal regions we created a gtf file with each entry corresponding to a unique TSS. We note that some genes are associated with more than one TSS; however, TEPIC by default only considers one entry for each unique gene symbol. Therefore, in order for TEPIC to treat these as separate regulatory regions (as we did in SPIDER), we listed a gene ("GeneName") that is annotated to multiple TSS as GeneName::COPY1, GeneName::COPY2, etc, one copy per unique TSS. When running TEPIC for proximal regions we set the window size to 1000, which corresponded to running TEPIC on regulatory regions defined as ±1kb around each TSS – the same regions we used for our proximal SPIDER analysis.

To run TEPIC using distal regions, we created a gtf file that had two entries for each unique TSS, one noting a location 22500bp upstream of the TSS and one noting a location 22500bp downstream of the TSS. These locations correspond to the mid-points of the regulatory regions we used when performing our distal analysis with SPIDER. In order for TEPIC to treat these as separate regulatory regions, each gene ("GeneName") was listed twice using the convention GeneName::U and GeneName::D, and annotated to the upstream and downstream TSS locations, respectively. As in the proximal analysis, genes that were associated with more than one TSS were denoted by GeneName::U::COPY1, GeneName::U::COPY2, etc. When running TEPIC for distal regions we set the window size to 2500, which corresponded to running TEPIC on regulatory regions located at ±20-25kb around each TSS – the same regions we used for our distal SPIDER analysis.

We ran TEPIC using DNase data for each of the six cell lines and gene annotations for proximal as well as distal regulatory regions (twelve total analyses). To handle multiple regulatory regions associated with the same gene (for example, a gene associated with multiple associated TSS and alternative 5' gene ends), for each TF motif to gene edge, we weighted the edge based on its maximum score across all possible inferences. Note that only genes associated with at least one regulatory region that overlaps with a DNase peak are contained in the TEPIC output file. Therefore, for completeness, edges targeting genes not in the TEPIC output were given a default score of zero; this ensured the networks we inferred using TEPIC contained the same set of target genes as the networks inferred using SPIDER.

Analyzing TEPIC-predicted networks: We first evaluated the accuracy of TEPIC predictions using continuous scores. Specifically, for each TF motif in each cell line, we extracted the vector of edge-weights from that motif to all genes in the TEPIC-predicted network and compared that vector to the TF's targets in the corresponding cell line specific ChIP-seq gold standard. Based on this analysis, we observed a mean AUC across all the tests of 0.7405 for the proximal analysis (**Supplemental Figure 5b-c**) and 0.7586 for the distal analysis (**Supplemental Figure 5e-f**). Overall the predictions made by TEPIC were more accurate than all the other methods/networks we evaluated, with the exception of SPIDER.

We also performed a threshold analysis. For each cell line network, we selected the top 25% of edges by weight. We evaluated the accuracy of each TF motif in these binarized networks using the corresponding TF and cell line specific ChIP-seq gold standard. Thresholding the TEPIC results negatively impacted its performance. When thresholding, we observe a mean AUC of only 0.5820 across all tests for the proximal analysis and a mean AUC of 0.6549 for the distal analysis. The results of this threshold analysis are shown in **Figure 5a** of the main text.

SPIDER: Our main evaluation of SPIDER's performance involved benchmarking the continuous edge scores predicted by SPIDER against ChIP-seq predictions. The results of these evaluations for individual TFs is shown in **Figures 2 & 4** (proximal and distal AUC, respectively) in the main text and in **Supplemental Figures 5a&c** (proximal and distal AUPR, respectively). These results are summarized across all cell lines and shown in **Supplemental Figure 5b-c** (proximal networks) and **Supplemental Figure 5e-f** (distal networks) alongside the subset of methods for which we could also perform continuous evaluations (six motifs in CENTIPEDE, PIQ, and TEPIC). For SPIDER, we observe a mean AUC of 0.7949 (proximal) and 0.8477 (distal) across all tests.

However, for many of the methods/networks described above we were only able to evaluate binary predictions. Therefore, to support a more equitable comparison of SPIDER to these other methods/networks, we also performed a threshold analysis based on SPIDER's predictions. As in the TEPIC evaluations (see above), for

each cell line network, we identified the top 25% of edges by weight. We evaluated the accuracy of each TF in these binarized networks using the corresponding TF and cell line specific ChIP-seq gold standard. We observed a mean AUC of only 0.6950 across all tests for the proximal analysis and a mean AUC of 0.7747 for the distal analysis. Although these AUC values are lower than the ones we obtained when evaluating SPIDER's continuous scores, they are still much higher than those we obtained from all other sources in the context of thresholding. The results of this threshold analysis are shown in **Figure 5a** of the main text.

SPIDER input data: For our final evaluation, we also determined the accuracy of the FIMO motif scan. This is an important evaluation since the quality of the data used to seed SPIDER may impact the algorithm's performance and explain its higher accuracy compared to the other sources we evaluated. To begin, we intersected the genomic locations of each motif with each of the ChIP-seq bed files corresponding to that motif's associated TF. Locations with an overlapping ChIP-seq peak were considered true binding events. Separately, we also intersected the genomic locations of each motif with open chromatin regions for each of the six cell lines, as defined in the DNase BED files used by SPIDER. Motif locations that overlapped with open chromatin regions were given a score of one, and all other locations were assigned a score of zero. This mirrors Step 1 of the SPIDER algorithm.

We used these data to calculate an AUC based on the initial PWM scores as well as based the binary "scores" obtained from overlapping with chromatin information. We found that, at a genome wide level, the data used as an input to SPIDER was already fairly accurate, with a mean AUC across all tests of 0.6596 based on PWM scores, and a mean AUC across all tests of 0.7431 based on the binary DNase-based scoring scheme. These results are illustrated in **Supplemental Figure 5a**. Overall, the accuracy of the motif scan that we used to construct the seed networks for SPIDER appears fairly similar to that used by CENTIPEDE and PIQ. Thus, the accuracy of the SPIDER input data cannot explain its highly superior performance compared to these other approaches in the gene regulatory network context.

Group 4: Algorithms that estimate networks using gene expression data

SPIDER uses chromatin accessibility data to construct transcription factor to gene networks. In contrast, most existing network reconstruction approaches focus on estimating these relationships using transcriptomic data. Although they rely on different input data, the goal of SPIDER is similar to that of these approaches. Therefore, we also compared SPIDER's performance to four commonly used expression-based network reconstruction methods: ARACNe (Algorithm for the Reconstruction of Accurate Cellular Networks)[8], CLR (Context Likelihood of Relatedness)[9], GENIE3 (GEne Network Inference with Ensemble of trees)[10], and PANDA (Passing Attributes between Networks for Data Assimilation)[11].

To begin, we identified lymphoblastoid gene expression data from the Genotype-Tissue Expression (GTEx) project; these data had previously been processed and used to build a lymphoblastoid regulatory network using PANDA[12]; 666 TFs and 19260 genes that were in our naïve seed network had measurable gene expression in these data. We used the expression data for these 666 TFs and 19260 genes as an input to ARACNe, CLR, GENIE3, and PANDA. GENIE3 and PANDA additionally allow the user to include input data describing an initial estimate of transcription factor to gene regulatory relationships. Therefore, for these two algorithms we included as an input a subset of the naive seed network -- the portion that extends between these 666 TFs and 19260 genes. To provide a fair comparison to SPIDER, we also re-ran SPIDER using only data pertaining to these same 666 TFs and 19260 genes. Finally, we benchmarked the five predicted networks (one each from ARACNe, CLR, GENIE3, PANDA, and SPIDER), using the associated portion of the ChIP-seq network for GM12878.

Overall, we find that using expression information to reconstruct a regulatory network is inferior to using epigenetic information (**Supplemental Figure 7**). The relatively low performance of the expression-based algorithms is likely due to two things: (1) gene expression represents a very noisy signal that needs to be assessed across multiple samples during network reconstruction; (2) these expression-based algorithms were primarily developed and benchmarked in the context of less complex organisms, such as *E. Coli* or yeast, or using *in silico* data. In contrast, SPIDER estimates a network using a single open-chromatin profile and has been optimized in the context of human data.

We note that upon close inspection, we find that SPIDER's performance seems to be slightly worse in this setting compared to what is reported in the main text of our manuscript. Specifically, the GM12878 SPIDER-predicted regulatory network has a reported AUC of 0.82 and AUPR of 0.42 in **Figure 2b**, but an AUC of 0.78 and AUPR of 0.42 in the analysis shown here. This is due to limiting the input data. In particular, our primary analyses use SPIDER to model regulatory interactions between all TFs and all genes. However, due to the requirements of the expression-based algorithms, the analyses described here use only a subset of these. The message-passing procedure used by SPIDER considers regulatory patterns across all TFs and genes when inferring a network. Thus, restricting the number of considered TFs and genes can hurt the algorithms' performance.

We would like to mention that we believe the inability to model non-expressed genes is a limitation of many expression-based network algorithms. Just because a gene doesn't have measurable expression doesn't mean it is not regulated. Namely, a gene can be targeted and repressed, causing it to have little to no measured expression. Including these lowly or non-expressed genes in PANDA and SPIDER is a strength of these algorithms.

S4. Potential of expanding SPIDER to incorporate other Omics data

SPIDER builds on the message passing framework used by the PANDA reconstruction algorithm. Thus, it has the potential to be extended to incorporate other sources of regulatory information, including protein-protein interaction and gene expression data.

To investigate this, we obtained protein-protein interaction (StringDB v10) and lymphoblastoid gene expression data from the Genotype-Tissue Expression (GTEx) project; these data had previously been processed and used to build a lymphoblastoid regulatory network using PANDA[12]. We removed transcription factors in the protein-protein interaction data and genes in the expression data that were not included in our naïve seed network. We then used these data together with the GM12878 DNase hypersensitivity data to generate various gene regulatory networks and benchmarked using the GM12878 "gold standard" ChIP-seq network; the results are shown **Supplemental Figure 8a**. More specifically, we first applied the PANDA algorithm to the protein-protein interaction data, gene expression data, and the naïve seed network used in our main text analysis. We see that in the absence of epigenetic information, PANDA is only able to slightly improve the accuracy of the naïve seed network, from 0.58 (naïve seed network) to 0.60 (PANDA regulatory network). We also observe an AUC of about 0.60 for the GM12878 SPIDER seed network, which represents the intersection of DNase data with the naïve seed network. The corresponding SPIDER regulatory network, which applies message-passing to SPIDER seed network has an AUC of 0.82. Next, we applied SPIDER to the GM12878 DNase hypersensitivity data, but included protein-protein interaction and gene co-expression information for P and C instead of identity matrices (see **Methods** in the main text). The final predicted network has an AUC of 0.80, which is slightly less than the SPIDER Regulatory Network.

To interpret this result, we investigated how the density of epigenetic information impacted the algorithm's performance. Specifically, we determined the degree (number of incoming edges) for the target genes in the SPIDER seed network. We then evaluated the accuracy of the edges targeting subsets of genes based on their degree (**Supplemental Figure 8b**). Although naively combining epigenetic, expression, and protein-protein interaction data using the PANDA/SPIDER framework did not directly result in a more accurate *overall* gene regulatory network, we observe a striking relationship between the density of information in the epigenetically informed SPIDER seed network and accuracy of the predictions. Specifically, adding protein interaction and gene expression data greatly improved the accuracy of edges targeting genes with low coverage in the SPIDER seed network (roughly, a degree less than 30). However, genes with relatively higher degree (from about 30 to 120 incoming edges) were more accurately predicted using only epigenetic data (SPIDER regulatory network). Genes that had an initial saturation of regulatory information (more than 120 incoming edges in the SPIDER seed network) also slightly benefitted from adding in gene expression and protein interaction data.

We believe these results are a consequence of the local density of the biological information that is coming from each Omics data source. In particular, it appears that the network structure induced by incorporating epigenetic information into the seed SPIDER network is highly informative for "medium degree" genes. For these genes, including expression data is equivalent to adding noise to the model, ultimately hurting edge predictions.

However, in the absence of sufficient information ("low degree" genes) – or a saturation of information ("high degree" genes), adding gene expression and protein-protein interaction data improves network predictions. Although promising, these results highlight the complexity of effectively integrating multiple sources of Omics data in a single methodological framework and points to the need for future systematic methods development in this area.

Supplemental Files:

Supplemental File 1: Information about data from ENCODE, **a.** A complete list of all ENCODE data files (DNase-Seq) used along with their locations on the ENCODE DCC website and other metadata. **b.** A complete list of all ENCODE data files (ChIP-seq) used along with their locations on the ENCODE DCC website.

Supplemental File 2: Supplemental Materials and Methods. Additional information regarding (S1) limitations when using transcription factor binding site predictions to construct gene regulatory networks; (S2) the performance of SPIDER when using random-like seed information; (S3) how we implemented or processed the data associated with other methods and available networks when comparing the performance of these sources with SPIDER; and (S4) an analysis exploring the potential to expand SPIDER to incorporate other Omics data.

Supplemental References:

1. Pique-Regi, R. et al. Accurate inference of transcription factor binding from DNA sequence and chromatin accessibility data. *Genome Res* **21**, 447-455 (2011).
2. Sherwood, R.I. et al. Discovery of directional and nondirectional pioneer transcription factors by modeling DNase profile magnitude and shape. *Nat Biotechnol* **32**, 171-178 (2014).
3. Cuellar-Partida, G. et al. Epigenetic priors for identifying active transcription factor binding sites. *Bioinformatics* **28**, 56-62 (2012).
4. Neph, S. et al. Circuitry and dynamics of human transcription factor regulatory networks. *Cell* **150**, 1274-1286 (2012).
5. Marbach, D. et al. Tissue-specific regulatory circuits reveal variable modular perturbations across complex diseases. *Nat Methods* **13**, 366-370 (2016).
6. Schmidt, F. et al. Combining transcription factor binding affinities with open-chromatin data for accurate gene expression prediction. *Nucleic Acids Res* **45**, 54-66 (2017).
7. Schmidt, F., Kern, F., Ebert, P., Baumgarten, N. & Schulz, M.H. TEPIC 2-an extended framework for transcription factor binding prediction and integrative epigenomic analysis. *Bioinformatics* **35**, 1608-1609 (2019).
8. Margolin, A.A. et al. ARACNE: an algorithm for the reconstruction of gene regulatory networks in a mammalian cellular context. *BMC Bioinformatics* **7 Suppl 1**, S7 (2006).
9. Faith, J.J. et al. Large-scale mapping and validation of Escherichia coli transcriptional regulation from a compendium of expression profiles. *PLoS Biol* **5**, e8 (2007).
10. Huynh-Thu, V.A., Irrthum, A., Wehenkel, L. & Geurts, P. Inferring regulatory networks from expression data using tree-based methods. *PLoS One* **5** (2010).
11. Glass, K., Huttenhower, C., Quackenbush, J. & Yuan, G.C. Passing messages between biological networks to refine predicted interactions. *PLoS One* **8**, e64832 (2013).
12. Sonawane, A.R. et al. Understanding Tissue-Specific Gene Regulation. *Cell Rep* **21**, 1077-1088 (2017).

Supplemental Tables:

Supplemental Table 1: Statistical evaluation of the increase in AUC for the SPIDER Regulatory Networks (SRN) compared to the naïve regulatory networks (NRN) and SPIDER seed networks (SSN). Density for the naïve seed (NSN) network was 39.73%.

Proximal Region (0 – 1kb from TSS)									
Cell line (SSN density)	AUC values (95% CI)			SRN vs NRN			SRN vs SSN		
	NRN	SSN	SRN	Percent increase	z-score (DeLong's test)	p-value	Percent increase	z-score (DeLong's test)	p-value (two-sided)
**A549 (5.89%)**	**0.574** (0.5722-0.5763)	**0.594** (0.5921-0.595)	**0.816** (0.8144-0.817)	42.1	-220.78	< 2.2e-16	37.3	-316.34	< 2.2e-16
**H1-HESC (6.04%)**	**0.597** (0.5957-0.5991)	**0.607** (0.6057-0.6083)	**0.726** (0.7248-0.7277)	21.6	-138.43	< 2.2e-16	19.6	-194.41	< 2.2e-16
**HELAS3 (5.64%)**	**0.579** (0.5725-0.5755)	**0.602** (0.6008-0.603)	**0.8093** (0.8072-0.8092)	39.7	-289.91	< 2.2e-16	34.3	-412.16	< 2.2e-16
**HEPG2 (5.49%)**	**0.583** (0.5778-0.5806)	**0.598** (0.5974-0.5994)	**0.770** (0.77-0.7721)	32.1	-248.52	< 2.2e-16	28.7	-344.19	< 2.2e-16
**GM12878 (5.77%)**	**0.581** (0.5771-0.5795)	**0.602** (0.6014-0.6031)	**0.819** (0.8178-0.8193)	40.9	-373.18	< 2.2e-16	36.0	-538.96	< 2.2e-16
**K562 (5.69%)**	**0.578** (0.5738-0.5761)	**0.596** (0.5956-0.5972)	**0.795** (0.7943-0.7959)	37.5	-358.64	< 2.2e-16	33.3	-505.68	< 2.2e-16

Supplemental Table 2: Characteristics of the input data as well as output predictions made for distal ranges.

**Ranges**	**GM12878**			**A549**		
	**AUC - SRN**	**AUC- SSN**	**Density (%)**	**AUC - SRN**	**AUC- SSN**	**Density (%)**
**±0-1kb**	0.819	0.602	5.77	0.815	0.593	5.89
**±5-10kb**	0.842	0.622	5.89	0.846	0.629	4.84
**±20-25kb**	0.855	0.620	4.97	0.849	0.627	4.73
**±45-50kb**	0.854	0.620	5.12	0.848	0.627	4.82
**±70-75kb**	0.853	0.620	5.10	0.846	0.624	4.77
**±95-100kb**	0.849	0.618	5.04	0.849	0.625	4.68

Supplemental Table 3: Statistical evaluation of the increase in AUC for the SPIDER Regulatory Networks (SRN) compared to the naïve regulatory networks (NRN) and SPIDER seed networks (SSN), based on a distal range of ±20-25kb around the TSS. Density for the naïve seed network (NSN) was 80.52%.

**Distal Region (-20 to -25kb and +20 to +25 kb from TSS)**									
Cell line (SSN density)	AUC values (95% CI)			SRN vs NRN			SRN vs SSN		
	NRN	SSN	SRN	Percent increase	z-score (DeLong's test)	p-value	Percent increase	z-score (DeLong's test)	p-value (two-sided)
**A549 (4.73%)**	**0.536** (0.5332-0.5382)	**0.628** (0.6258-0.6298)	**0.85** (0.8478-0.8514)	58.58	-196.17	< 2.2e-16	35.32	-218.68	< 2.2e-16
**H1-HESC (5.36%)**	**0.513** (0.4844-0.4887)	**0.625** (0.6232-0.6268)	**0.807** (0.8056-0.8089)	57.30	-225.91	< 2.2e-16	29.16	-214.0	< 2.2e-16
**HELAS3 (5.16%)**	**0.503** (0.5043-0.5083)	**0.615** (0.6135-0.6165)	**0.846** (0.8442-0.8467)	68.2	-271.35	< 2.2e-16	37.47	-317.46	< 2.2e-16
**HEPG2 (4.76%)**	**0.526** (0.517-0.5205)	**0.614** (0.6131-0.6156)	**0.803** (0.8014-0.8041)	52.66	-260.54	< 2.2e-16	30.7	-277	< 2.2e-16
**GM12878 (4.97%)**	**0.522** (0.4826-0.4857)	**0.62** (0.6192-0.6216)	**0.855** (0.8538-0.8557)	63.79	-382.33	< 2.2e-16	37.9	-401.31	< 2.2e-16
**K562 (5.56%)**	**0.495** (0.5077-0.5107)	**0.616** (0.615-0.6172)	**0.832** (0.8307-0.8326)	68.08	-354.71	< 2.2e-16	35.06	-414.83	< 2.2e-16

Supplemental Table 4: Results when evaluating predicted TF–gene relationships estimated or provided by various sources. The number of tests performed for each source are listed as well as the mean AUC and AUPR values across all tests. Note that not all evaluations could be performed in all sources. In particular, continuous AUC and AUPR could not be calculated for many sources. CENTIPEDE-sub refers to a small subset of CENTIPEDE predictions for which we had continuous prediction information. CL=cell line; TF=transcription factor; thresh = threshold analysis; cont = continuous analysis; PWM = position weight matrix score used; * = sources shown in **Figure 5**; † = sources shown in **Supplemental Figure 5d**.

	**Characteristics**			**Average AUC Across Tests**						**Average AUPR Across Tests**			
	# Evaluated			proximal		distal		genome-wide		proximal	distal	genome-wide	
**Sources**	TFs	CLs	Tests	thresh	cont	thresh	cont	PWM	Score	cont	cont	PWM	Score
CENTIPEDE-sub	6	1	6	0.524	0.540	0.519	0.563	0.704	0.978	0.316	0.199	0.303	0.730
CENTIPEDE*†	19	5	47	0.516	X	0.522	X	X	X	X	X	X	X
PIQ*†	25	1	48	0.556	0.570	0.539	0.561	0.600	0.773	0.280	0.141	0.066	0.229
Neph*	64	2	91	0.518	X	X	X	X	X	X	X	X	X
Marbach*	74	5	167	0.559	X	X	X	X	X	X	X	X	X
TEPIC*†	97	6	243	0.582	0.741	0.655	0.759	X	X	0.284	0.221	X	X
SPIDER*†	103	6	254	0.695	0.795	0.775	0.848	X	X	0.374	0.332	X	X
SPIDER Input	103	6	254	0.601	X	0.622	X	0.660	0.743	X	X	X	X
Figure #	X	X	X	5	S5b	S5d	S5e	S5a	S5a	S5c	S5f	X	X

Supplemental Table 5: The frequency of top genes in significant SPIDER edges (FDR<0.05) that were not part of the SPIDER seed network, for each of the six cell lines and for the proximal and distal regions.

Cell line	Most frequent genes among novel edges inferred (FDR p<0.05) by SPIDER			
	**Proximal region (±0-1kb)**		**Distal region (±20-25kb)**	
A549	PDE4D	200	MIR6870	378
	MIR100HG	132	GPR25	375
	ZBTB20	108	CABLES1	369
	TGIF1	85	NINJ2	369
	LINC-PINT	31	SEPT9	368
	NCOA7	29	NFKBIZ	366
	MIA2	11	MIR100HG	362
	ABLIM1	9	SERTAD3	362
	FAM107B	5	MIR663B	360
H1-HESC	No significant edges		CXXC5	241
			HOXA3	211
			SEPT9	209
			BDNF	134
			FGR	116
HELA-S3	MIA2	163	PPARG	297
	ZBTB20	139	PLEC	286
	PLEC	35	CASC21	273
	MIR99AHG	25	KRT8	271
	UBE2D3	2	SMAD3	267
	CXXC5	1	PDE4D	257
	DNAJB4	1	CASC19	253
HEPG2	No significant edges		CFLAR	288
			NDUFS2	271
			SYS1	268
			CXXC5	205
			PCBD2	203
			FAM50B	199
			KIFC3	197
GM12878	SEPT9	106	SLC44A2	376
	ZFP36L1	2	LOC100120357	375
			TMEM151B	375
			MXRA7	373
			SPTBN1	372
K562	FOXP1	84	CXXC5	290
	MAPRE2	1	MIR4761	281
	SAMSN1	1	PHF19	277
			COMT	268
			RNY4	262

Supplemental Table 6: For each of the six cell lines, the top edges (by weight) that were predicted by SPIDER around TFs for which we had ChIP-seq data in that cell line. Top edges that either had, or did not have, motif evidence are shown. Light blue shading indicates a true positive in the seed network that was retained after running SPIDER; light red shading indicates a false negative in the seed network that was recovered by SPIDER.

	Cell line	Range	TF	Gene	SRN	Motif	ChIP-seq
1.	A549	0-1kb	MAX	MIR100HG	22.35	Yes	No
			CEBPB	PDE4D	21.07	Yes	Yes
			SIX5	PDE4D	20.22	Yes	Yes
			CREB1	PDE4D	10.03	No	Yes
			ETS1	MIR100HG	8.86	No	No
			MAX	PDE4D	6.86	No	Yes
	A549	20- 25kb	MAX	PDE4D	38.50	Yes	Yes
			ETS1	PDE4D	34.85	Yes	Yes
			CREB1	PDE4D	32.49	Yes	Yes
			YY1	PDE4D	14.61	No	No
			ETS1	SMAD3	12.85	No	Yes
			CREB1	ITGB1	12.25	No	Yes
2.	H1HESC	0-1kb	SP1	CNGA4	19.69	Yes	No
			SRF	FOXP1	18.86	Yes	Yes
			SP1	SUGT1P1	18.77	Yes	No
			ZNF143	PAX6	3.76	No	Yes
			SIX5	PAX6	3.48	No	No
			BACH1	PAX6	3.22	No	Yes
	H1-HESC	20-25kb	SRF	CXXC5	25.12	Yes	No
			MAX	CXXC5	23.53	Yes	No
			SRF	HOXA3	22.12	Yes	Yes
			TEAD4	CXXC5	8.44	No	Yes
			CEBPB	CXXC5	8.43	No	No
			NANOG	HOXA3	8.29	No	No
3.	HELA-S3	0-1kb	E2F4	MIA2	24.76	Yes	No
			E2F1	MIA2	21.28	Yes	No
			MAZ	TRANK1	19.75	Yes	No
			E2F1	ZBTB20	6.96	No	Yes
			ZNF143	MIA2	6.51	No	No
			TCF7L2	MIA2	6.18	No	No
	HELA-S3	20-25kb	E2F4	KRT8	29.19	Yes	No
			RFX5	PDE4D	26.52	Yes	Yes
			STAT1	PDE4D	26.19	Yes	Yes
			E2F1	PDE4D	13.12	No	No
			ZNF143	PDE4D	11.90	No	No
			MAX	PDE4D	11.77	No	Yes
4.	HEPG2	0-1kb	MYBL2	FOXP1	21.91	Yes	No
			MAZ	DKFZP434H168	20.12	Yes	No
			SRF	PDE4DIP	19.78	Yes	No
			HSF1	FOXP1	4.97	No	No

			HSF1	MIA2	4.18	No	No
			HSF1	MIR4461	4.17	No	No
	HEPG2	20-25kb	BRCA1	CFLAR	28.18	Yes	No
			SRF	CFLAR	27.36	Yes	Yes
			HSF1	CFLAR	25.10	Yes	No
			MYBL2	CFLAR	10.32	No	Yes
			SRF	NDUFS2	8.02	No	Yes
			SRF	SYS1	7.97	No	No
5.	GM12878	0-1kb	MAX	SEPT9	24.35	Yes	Yes
			STAT5A	SEPT9	20.15	Yes	Yes
			EBF1	SEPT9	20.07	Yes	Yes
			IKZF1	SEPT9	7.6	No	No
			ETS1	SEPT9	6.24	No	Yes
			ELK1	SEPT9	5.55	No	No
		20-25kb	ELK1	CXXC5	30.49	Yes	Yes
			SRF	CXXC5	30.26	Yes	No
			STAT3	CXXC5	28.86	Yes	Yes
			IKZF1	CXXC5	15.07	No	Yes
			SIX5	CXXC5	14.46	No	No
			PAX5	CXXC5	14.15	No	Yes
6.	K562	0-1kb	SRF	FOXP1	22.86	Yes	No
			ZNF263	SKAP1	19.19	Yes	No
			MAZ	DKFZP434H168	19.18	Yes	Yes
			ZNF143	FOXP1	6.47	No	No
			ETS1	FOXP1	6.22	No	No
			THAP1	FOXP1	6.15	No	No
		20-25kb	MAX	CXXC5	27.59	Yes	Yes
			ETS1	COMT	26.68	Yes	Yes
			ELK1	CXXC5	26.34	Yes	Yes
			STAT5A	COMT	10.52	No	Yes
			MAX	COMT	10.41	No	Yes
			E2F4	CXXC5	10.14	No	Yes

<u>Supplemental Figures:</u>

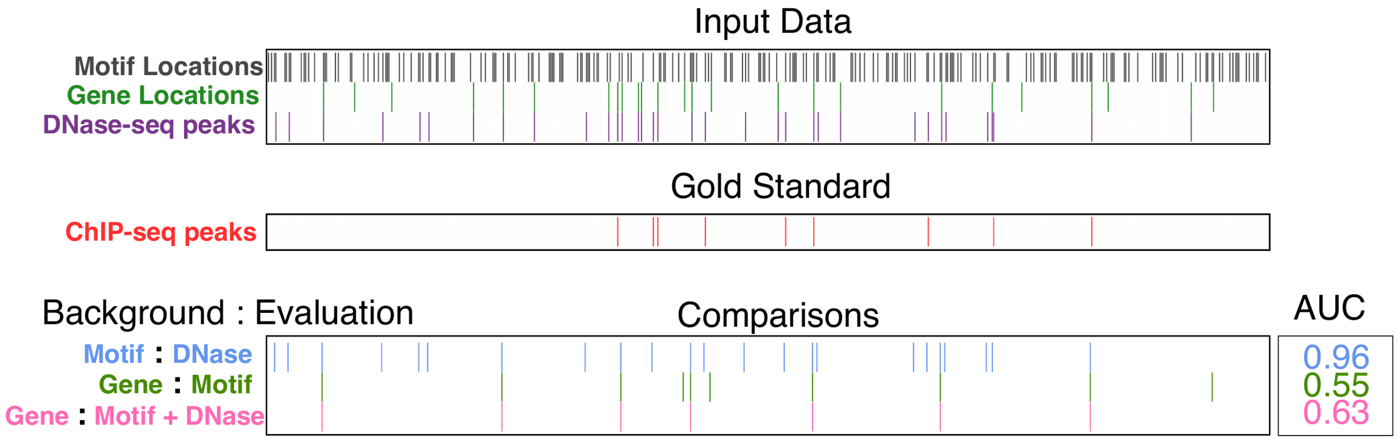

Supplemental Figure 1: Illustrative example of how transcription factor binding site predictions can be used to create a seed regulatory network, and the limited impact of chromatin information on network accuracy. The top panel shows the genomic locations of transcription factor motifs, gene promoters, and open chromatin regions; gene promoters are more likely to be in regions of open chromatin. The second panel shows the true locations of transcription factor binding based on ChIP-seq data. The bottom panel illustrates combinations of data from the top panel. These combinations were evaluated using a benchmark derived from the ChIP-seq data in the middle panel. The term to the left of the colon indicates the set of elements being evaluated, while the term to the right indicates what is considered a positive prediction. For example, in the final row, each *gene* is assigned a value of 1 or 0 based on whether or not that gene's promoter also contains both a *motif* and a *DNase*-seq peak; this is benchmarked against information regarding whether or not each gene's promoter contains a true binding event, based on ChIP-seq peaks. The AUC value in this evaluation is 0.63. See also **Supplemental Section S1**.

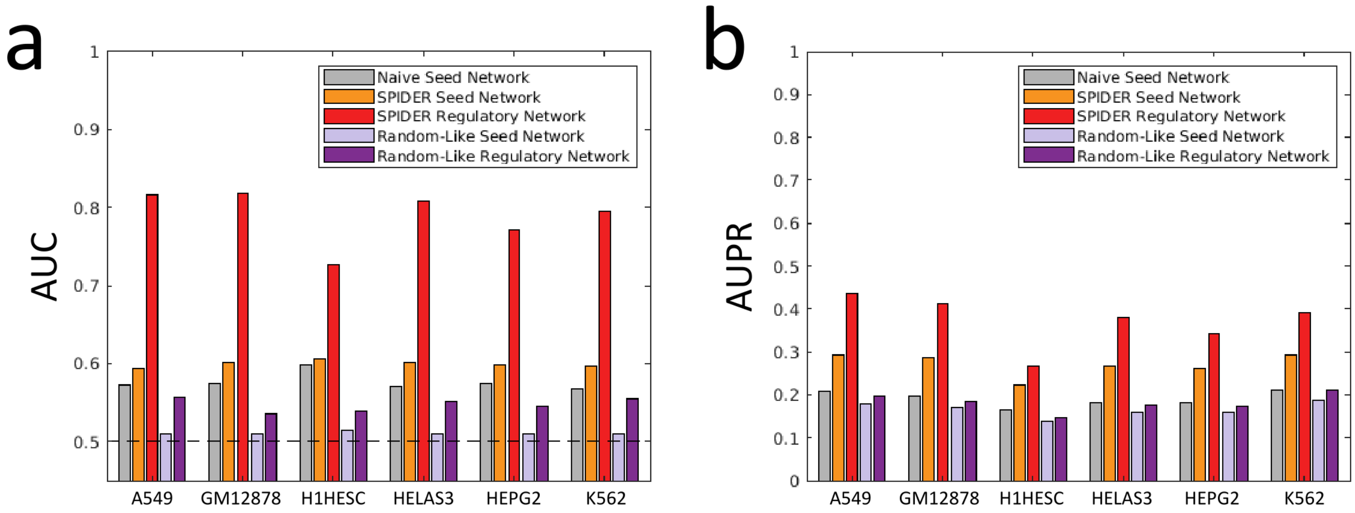

Supplemental Figure 2: Application of message-passing to random-like seed networks. The AUC and AUPR values for the naïve seed, SPIDER seed, and SPIDER regulatory network in each cell line, as shown in **Figure 2**, as well as the mean AUC and AUPR values across the random-like seeds and their corresponding random-like regulatory networks. Random-like seeds for each cell line were constructed by randomly pruning edges from the naïve seed network to match the density of the SPIDER seed networks in that cell line.

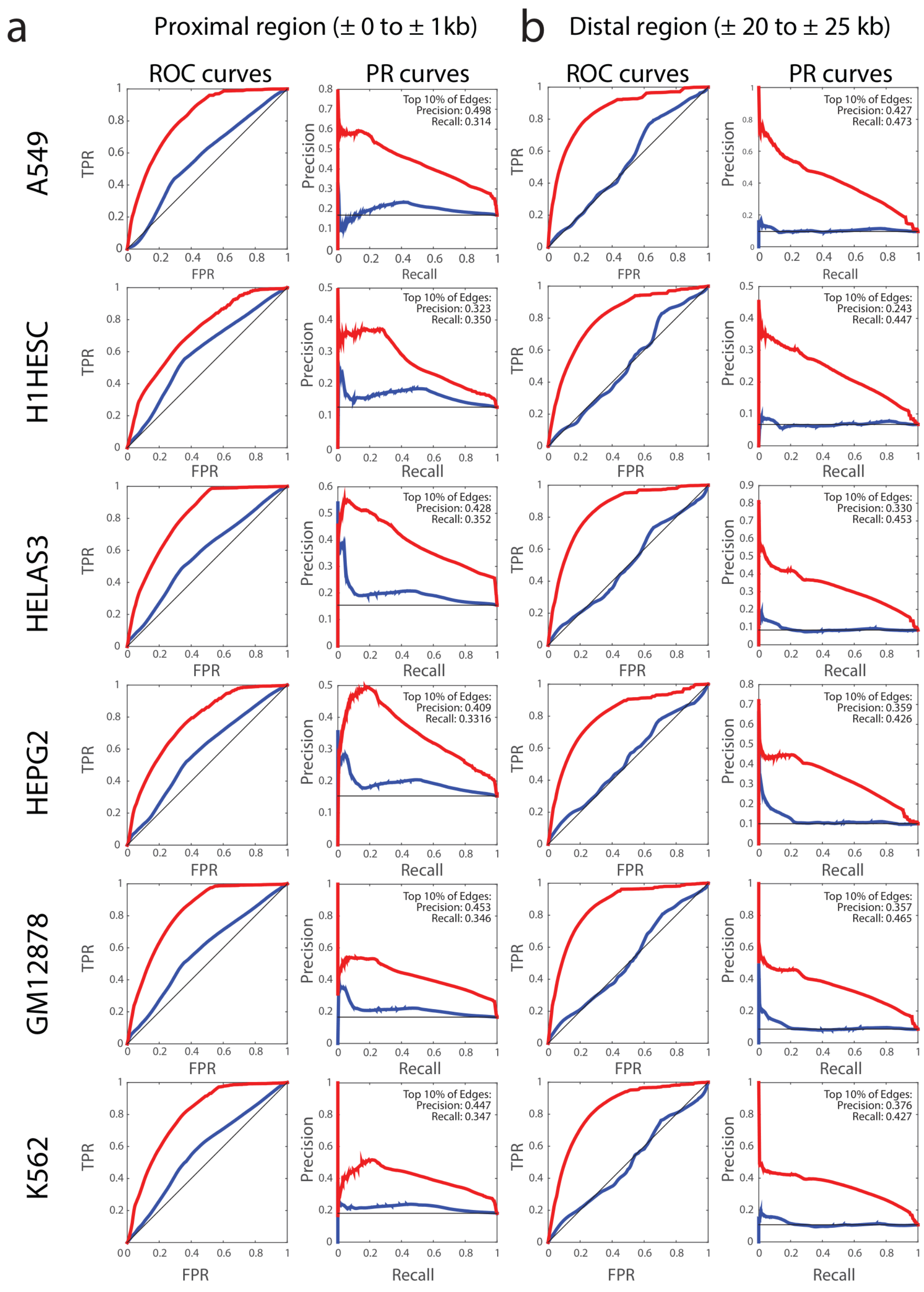

Supplemental Figure 3: Receiver-Operating Characteristic (ROC) and Precision-Recall (PR) curves. ROC and PR curves for the naïve regulatory network (blue) and SPIDER regulatory network (red), evaluated using ChIP-seq data from six cell lines. The precision and recall values obtained when selecting the top 10% of edges is also noted. Results are shown for both the **a.** proximal regulatory networks and **b.** distal regulatory models. See also **Figure 2**, **Figure 4**, **Supplemental Table 1**, and **Supplemental Table 3**.

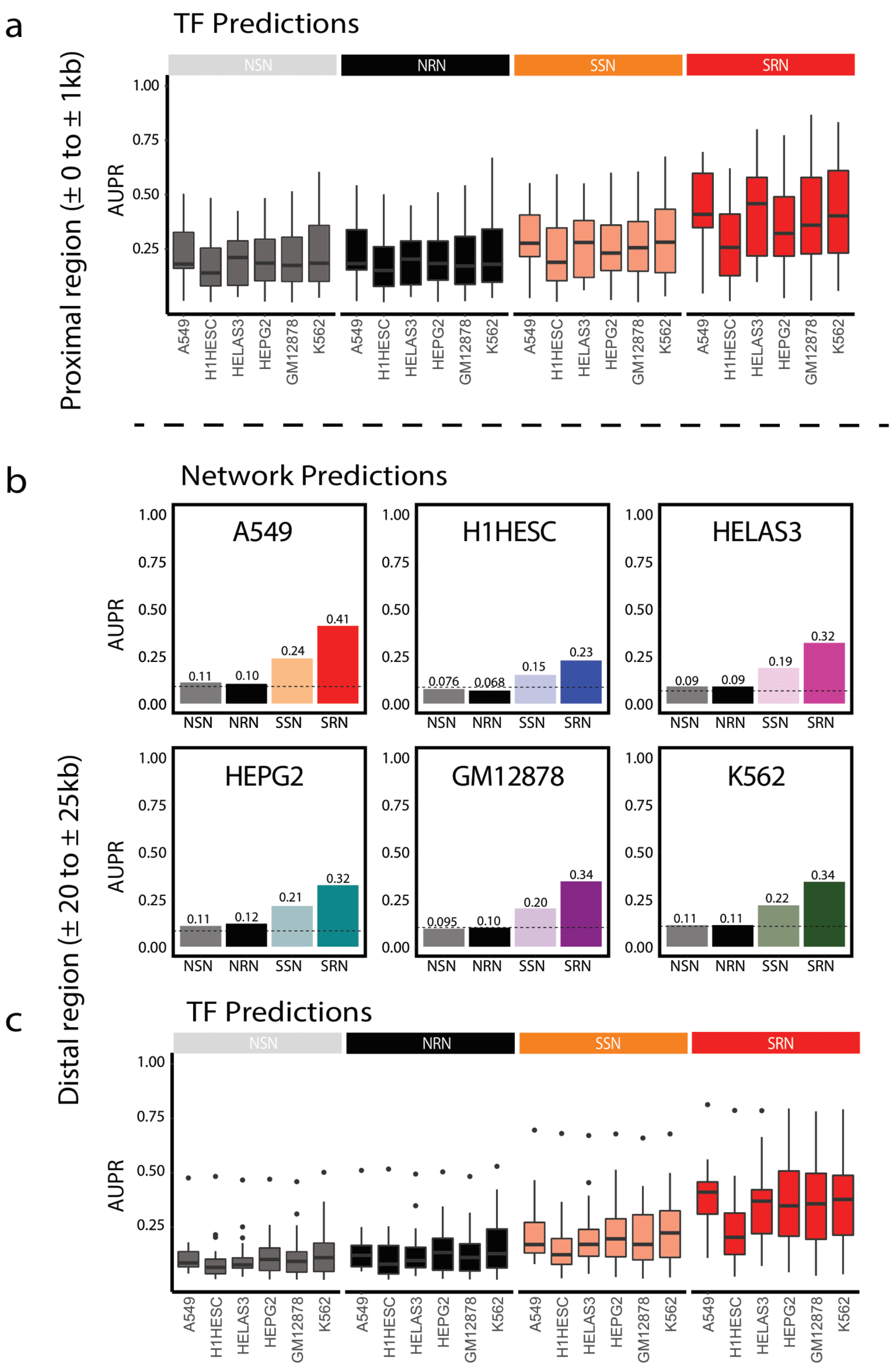

Supplemental Figure 4: AUPR values for the proximal and distal regulatory predictions. a. Distribution of AUPR scores across individual transcription factors for four types of proximal networks in six cell lines. **b.** AUPR scores for four types of distal regulatory models in six cell lines. **c.** Distribution of AUPR scores across individual transcription factor predictions in the distal regulatory models. Box plot elements: center line: median; box limits: 1st and 3rd quartiles; whiskers: upper and lower fence; points: outliers. See also **Figure 2** and **Figure 4**.

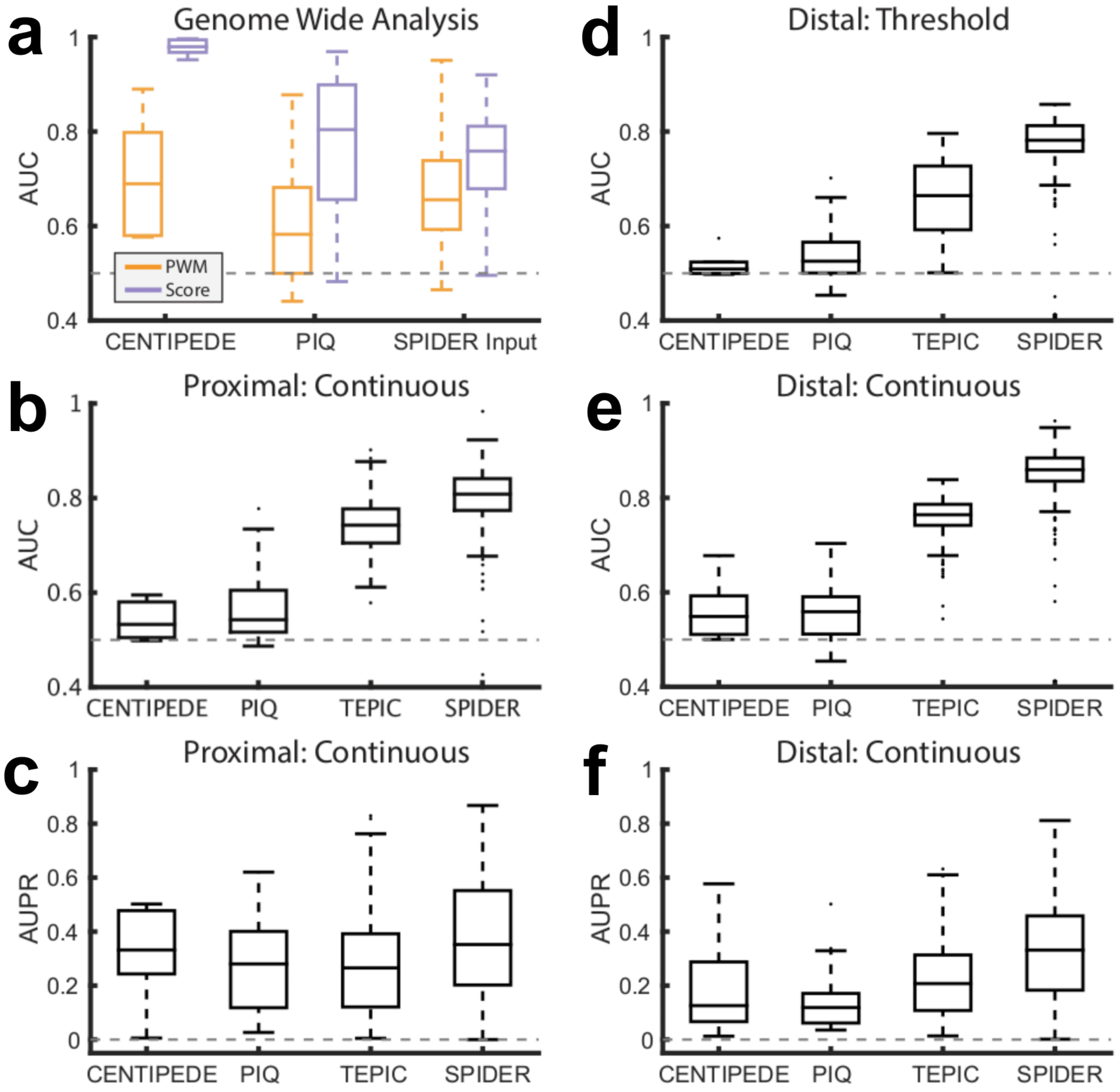

Supplemental Figure 5: Detailed comparative benchmarking of SPIDER with other methods. a. Performance of CENTIPEDE, PIQ, and the input to SPIDER (the overlap of DNase peaks with motif predictions), when all transcription factor motif locations across the genome are considered in the evaluation, as measured by AUC. **b-c**. Performance of the networks derived from the output of CENTIPEDE and PIQ, as well as the networks predicted by TEPIC and SPIDER, based on continuous (as opposed to thresholded) edge-weights, as measured by (**b**) AUC and (**c**) AUPR. **d-f**. Performance of the networks derived from the output of CENTIPEDE and PIQ, as well as the networks predicted by TEPIC and SPIDER, using distal regulatory regions, based on (**d**) thresholded and (**e-f**) continuous edge-weights, and evaluated using the (**d-e**) AUC and (**f**) AUPR. These analyses demonstrate that even though some methods, such as CENTIPEDE and PIQ, perform well at predicting ChIP-seq binding when scoring motif locations (as shown in panel **a**), this does not translate into accurate network-level predictions (as demonstrated in panels **b-f**). Box plot elements: center line: median; box limits: 1st and 3rd quartiles; whiskers: upper and lower fence; points: outliers. See also **Supplemental Section S1**, **Supplemental Figure 1**, **Supplemental Table 4**, and **Figure 5**.

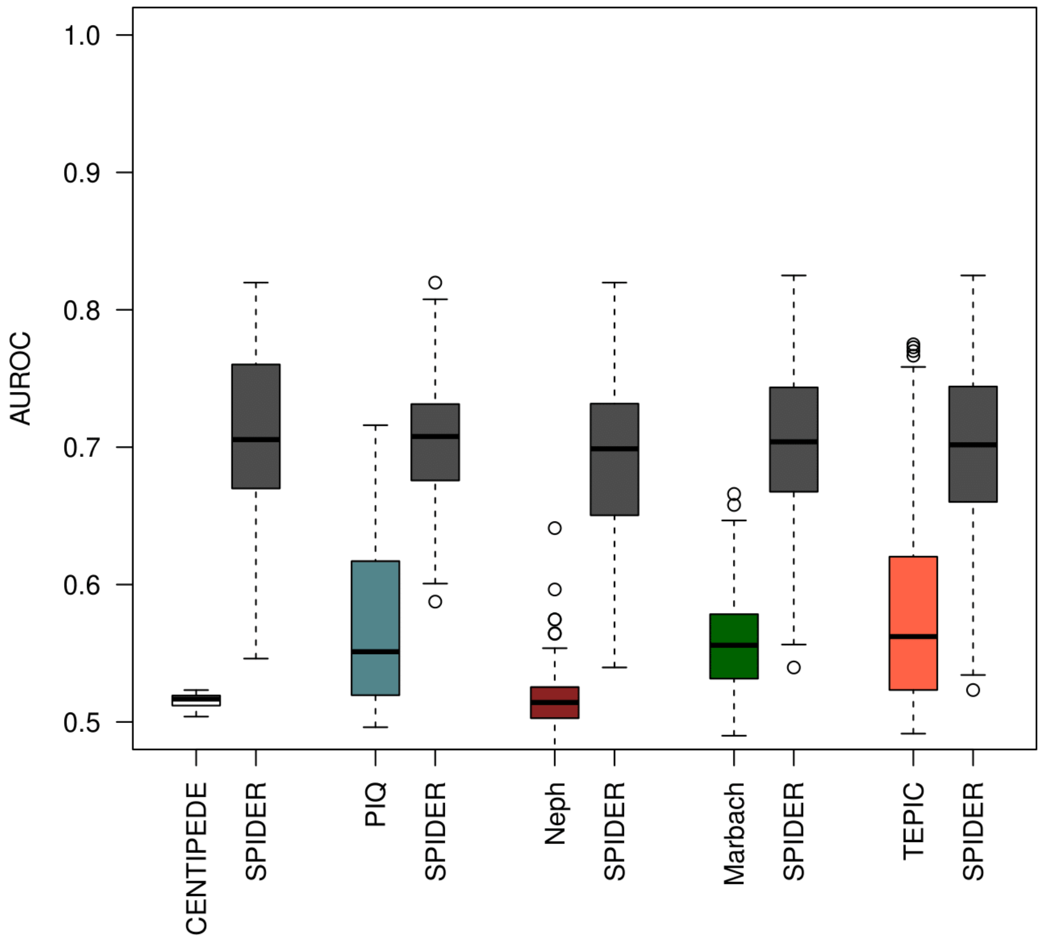

Supplemental Figure 6: Performance of SPIDER compared to other methods when restricting to tests performed for both methods. The distribution of AUC values for the tests performed (transcription factor / cell line pairs) for each method, and the distribution of AUC values for those same tests using the results predicted by SPIDER. The distribution of values for each method is identical to those shown in **Figure 5** but varies for SPIDER depending on the selected tests. Box plot elements: center line: median; box limits: 1st and 3rd quartiles; whiskers: upper and lower fence; points: outliers. See also **Supplemental Table 4**.

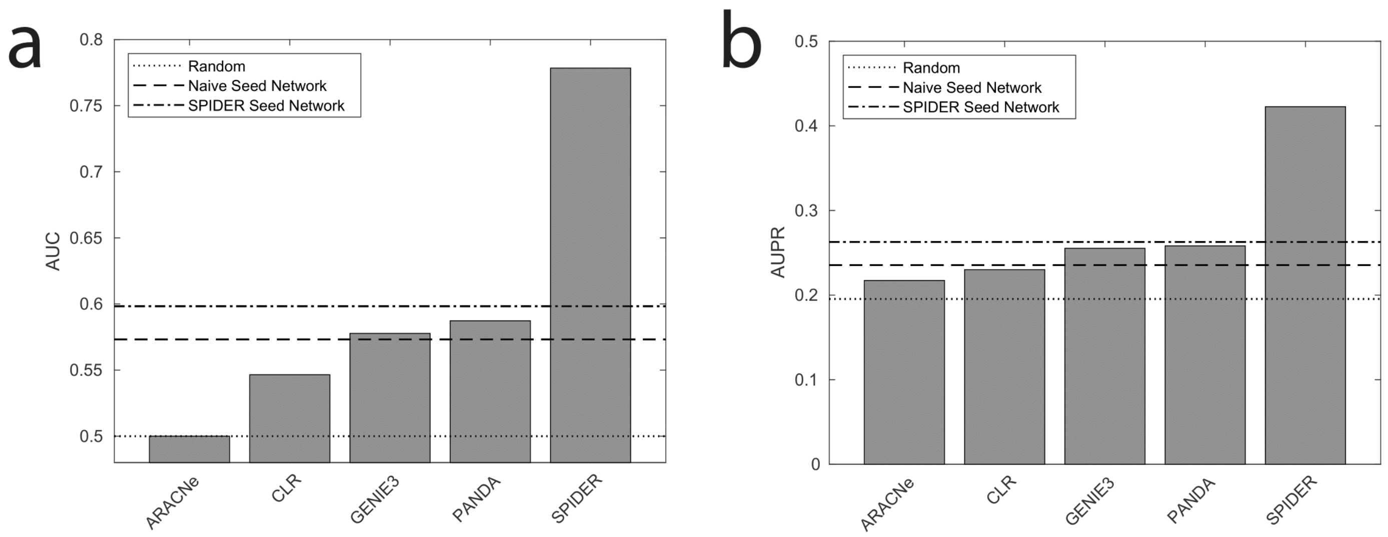

Supplemental Figure 7: Performance of SPIDER compared to expression-based network inference algorithms. Performance of ARACNe, CLR, GENIE3, PANDA, and SPIDER in terms of **a.** AUC and **b.** AUPR. Gene expression data is used as an input to ARACNe, CLR, GENIE3, and PANDA. GENIE3 and PANDA also take as input data prior information regarding potential transcription factor to gene interactions (naïve seed network, illustrated as a dashed line). In contrast SPIDER uses open chromatin data to construct a seed network (dash-dotted line) that is then refined using message-passing. Due to the restrictions of the expression-based network reconstruction approaches, the networks reconstructed in this analysis only include information for a subset of transcription factors and genes. See also **Supplemental Section S3**.

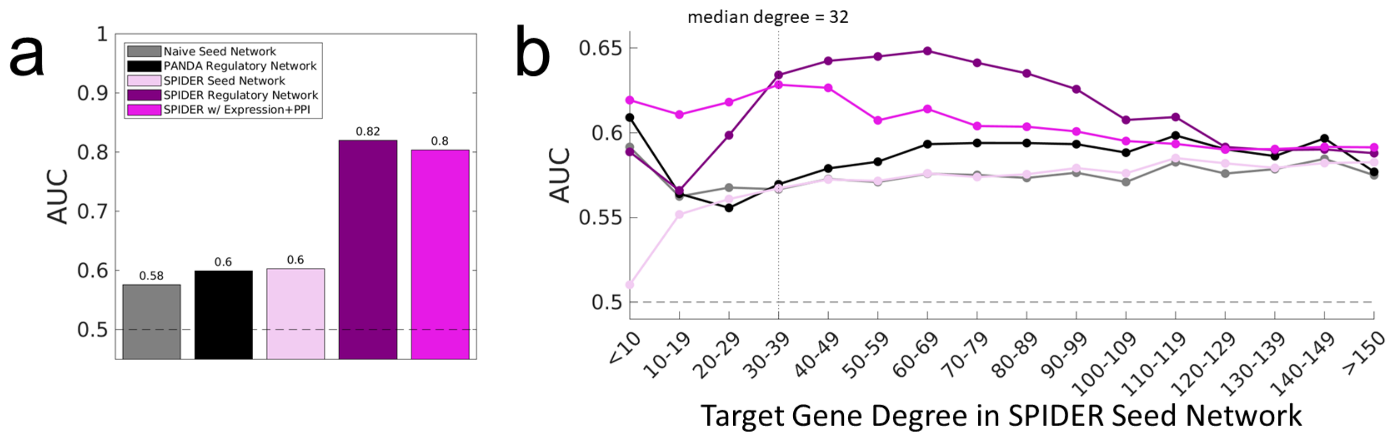

Supplemental Figure 8: Comparing SPIDER and PANDA a. The accuracy of the overall networks predicted using PANDA or SPIDER with various combinations of input data. **b.** The accuracy of non-overlapping subsets of these networks, specifically, all possible edges targeting genes that have a specific degree-range in the SPIDER seed network. The median degree of 32 is noted by a vertical dotted line.

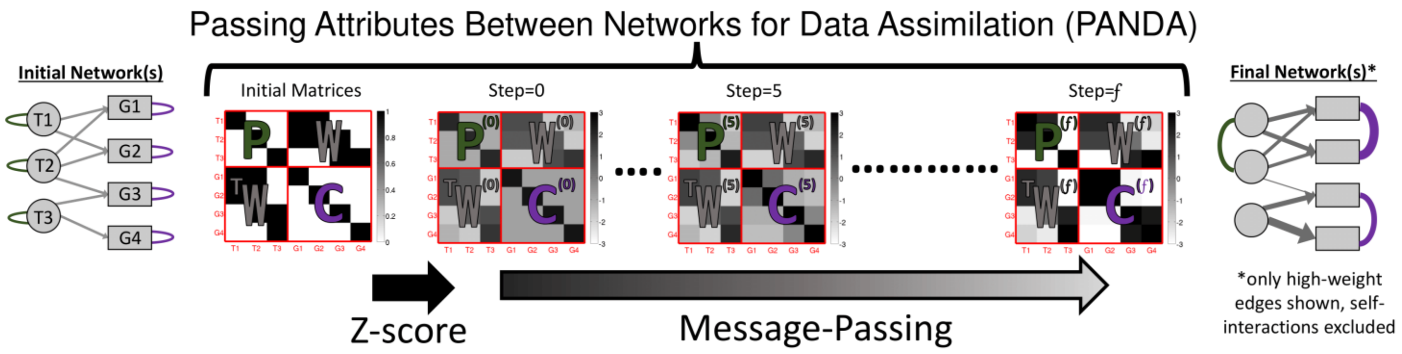

Supplemental Figure 9: Illustration of the PANDA message-passing procedure.
